# Changes in subcutaneous white adipose tissue cellular composition and molecular programs underlie glucose intolerance in persons with HIV

**DOI:** 10.3389/fimmu.2023.1152003

**Published:** 2023-08-30

**Authors:** Samuel S. Bailin, Jonathan A. Kropski, Rama D. Gangula, LaToya Hannah, Joshua D. Simmons, Mona Mashayekhi, Fei Ye, Run Fan, Simon Mallal, Christian M. Warren, Spyros A. Kalams, Curtis L. Gabriel, Celestine N. Wanjalla, John R. Koethe

**Affiliations:** ^1^ Department of Medicine, Division of Infectious Diseases, Vanderbilt University Medical Center, Nashville, TN, United States; ^2^ Department of Medicine, Division of Allergy, Pulmonary, and Critical Care Medicine, Vanderbilt University Medical Center, Nashville, TN, United States; ^3^ Veterans Affairs Tennessee Valley Healthcare System, Nashville, TN, United States; ^4^ Deparment of Cell and Developmental Biology, Vanderbilt University, Nashville, TN, United States; ^5^ Tennessee Center for AIDS Research, Vanderbilt University Medical Center, Nashville, TN, United States; ^6^ Department of Medicine, Division of Diabetes, Endocrinology, and Metabolism, Vanderbilt University Medical Center, Nashville, TN, United States; ^7^ Department of Biostatics, Division of Epidemiology, Vanderbilt University Medical Center, Nashville, TN, United States; ^8^ Department of Biostatistics, Vanderbilt University Medical Center, Nashville, TN, United States; ^9^ Insitute for Immunology and Infectious Diseases, Murdoch University, Perth, WA, Australia; ^10^ Vanderbilt Technologies for Advanced Genomics, Vanderbilt University Medical Center, Nashville, TN, United States; ^11^ Center for Translational Immunology and Infectious Diseases, Vanderbilt University Medical Center, Nashville, TN, United States; ^12^ Department of Medicine, Division of Gastroenterology, Hepatology, and Nutrition, Nashville, TN, United States

**Keywords:** human immunodeficiency virus, type 2 diabetes mellitus, white adipose tissue, subcutaneous adipose tissue, single-cell RNA sequencing, immune cells, glucose intolerance

## Abstract

**Introduction:**

Subcutaneous adipose tissue (SAT) is a critical regulator of systemic metabolic homeostasis. Persons with HIV (PWH) have an increased risk of metabolic diseases and significant alterations in the SAT immune environment compared with the general population.

**Methods:**

We generated a comprehensive single-cell multi-omic SAT atlas to characterize cellular compositional and transcriptional changes in 59 PWH across a spectrum of metabolic health.

**Results:**

Glucose intolerance was associated with increased lipid-associated macrophages, CD4^+^ and CD8^+^ T effector memory cells, and decreased perivascular macrophages. We observed a coordinated intercellular regulatory program which enriched for genes related to inflammation and lipid-processing across multiple cell types as glucose intolerance increased. Increased CD4^+^ effector memory tissue-resident cells most strongly associated with altered expression of adipocyte genes critical for lipid metabolism and cellular regulation. Intercellular communication analysis demonstrated enhanced pro-inflammatory and pro-fibrotic signaling between immune cells and stromal cells in PWH with glucose intolerance compared with non-diabetic PWH. Lastly, while cell type-specific gene expression among PWH with diabetes was globally similar to HIV-negative individuals with diabetes, we observed substantially divergent intercellular communication pathways.

**Discussion:**

These findings suggest a central role of tissue-resident immune cells in regulating SAT inflammation among PWH with metabolic disease, and underscore unique mechanisms that may converge to promote metabolic disease.

## 1 Introduction

The rising global rates of obesity and type 2 diabetes mellitus (T2DM) represent a significant public health challenge (1, 2). Persons with HIV (PWH) suffer disproportionately from cardiovascular disease, chronic kidney disease, and T2DM compared with the general population (3–6). The higher risk of metabolic disease in PWH compared with the general population is likely multifactorial and stems from increasing age and survival with improved antiretroviral medications, a greater prevalence of known risk factors for metabolic disease including elevated body mass index (BMI), and HIV-specific risk factors including persistent inflammation (3, 7–9). Adipose tissue is an important regulator of glucose and lipid metabolism (10), and alterations in its cellular composition and function have been implicated in the development of metabolic disease (11–15). While alterations in adipose tissue are common in PWH (7), the underlying changes in subcutaneous adipose tissue (SAT) that contribute to metabolic disease in this population are poorly understood.

Despite the success of modern antiretroviral therapy (ART), PWH with sustained suppression of plasma viremia still have persistent innate and adaptive immune activation (16), as well as lasting alterations in the adipose tissue immune cell compartment (17). In non-obese PWH, CD8^+^ T cells accumulate in SAT in a process strikingly similar to that observed in obesity in the general population (18, 19). Additionally, SAT CD4^+^ T cells in PWH shift towards an increased proportion of inflammatory and cytotoxic cells compared to HIV-negative individuals (20). Many immune cell populations implicated in the perpetuation of chronic SAT inflammation in studies of HIV-negative individuals or animals are also altered in circulating immune cells in PWH (21, 22). Recent studies in lean and obese persons in the general population have begun to unravel the complex cellular composition of adipose tissue (14, 15, 23, 24). However, a comprehensive assessment of the identity, polarization, and molecular programs of adipose tissue immune cells in PWH, a group at particularly high risk of metabolic disease, has not been reported.

We recruited a large cohort of PWH with a spectrum of metabolic health to elucidate the SAT cellular compositional and transcriptional regulatory framework that defines metabolic disease in this population. We used single-cell proteogenomics and generated a detailed molecular atlas of SAT from 59 PWH to characterize disease-specific changes in cellular populations and expression programs. In PWH with glucose intolerance, we found higher proportion of lipid-associated macrophage (LAM) and LAM-like macrophage populations, reduction of perivascular macrophages (PVMs), and an increase in CD4^+^ and CD8^+^ T effector memory (T_EM_) populations that was independent of obesity, age, and sex. We further uncovered a multicellular transcriptional regulatory program with glucose intolerance characterized by a shift towards macrophage lipid processing phenotype, increasing cytotoxicity and IFN-γ phenotype in T cells, and genes associated with fibrosis. Intercellular communication analysis predicted increased signaling of several inflammatory and pro-fibrotic pathways among PWH with glucose intolerance compared with non-diabetic PWH. Finally, we show that while the SAT cellular composition is broadly similar between diabetic PWH and diabetic HIV-negative individuals, there are important differences in cell-signaling that influence macrophage polarization, extracellular matrix deposition, and adipogenesis, and may affect disease severity. These transcriptomic and other data are publicly available on a user-friendly interactive website (http://vimrg.app.vumc.org/) for the research community.

## 2 Materials and methods

### 2.1 Study participants

Participants were members of the HIV, Adipose Tissue Immunology, and Metabolism (HATIM) study developed to evaluate adipose tissue characteristics in the context of HIV infection and metabolic disease (ClinicalTrials.gov registration NCT04451980). PWH were recruited from the Vanderbilt University Medical Center Comprehensive Care Clinic between August 2017 and June 2018, were on antiretroviral therapy (ART) for ≥ 18 months, had virologic suppression (serum HIV-1 RNA quantification < 50 copies/mL) for ≥ 12 months, had a CD4^+^ T cell count ≥ 350 cells/mm^3^ and had no known inflammatory or rheumatologic conditions. Participants were classified as non-diabetic (hemoglobin A1c [HbA1c] < 5.7% and/or fasting blood glucose [FBG] < 100 mg/dL), prediabetic (HbA1c 5.7-6.4% and/or FBG 100-125 mg/dL), or diabetic (HbA1c > 6.4% and/or FBG ≥ 126 mg/dL and/or on anti-diabetic medication) in accordance with the American Diabetes Association criteria (25). HIV-negative participants with diabetes were simultaneously recruited from the Vanderbilt *ResearchMatch* cohort, and these individuals were group matched by age and BMI with diabetic PWH. All participants underwent a single clinical research visit after a minimum 8-hour fast, including HbA1c and FBG measurement, peripheral blood mononuclear cell (PBMC) collection, fasting plasma collection, and anthropomorphic measurements. All individuals also underwent subcutaneous adipose tissue liposuction as described below. The Vanderbilt Institutional Review Board approved the research (IRB # 161254), and all participants provided written consent.

### 2.2 Adipose tissue collection and processing

Subcutaneous adipose tissue biopsies were collected approximately 3 centimeters to the right of the umbilicus after anesthetizing the skin with lidocaine/epinephrine and infiltrating 40 mL of sterile saline and lidocaine into the SAT. We collected approximately 5 grams of adipose tissue using a 2.1 mm blunt, side-ported liposuction catheter (Tulip CellFriendly**™** GEMS system Miller Harvester, Tulip Medical Products) designed for extraction of viable adipocytes and stromal vascular fraction (SVF) during cosmetic adipose tissue transfer procedures (26). Using this method, adipose tissue is recovered in tissue fragments generally < 3 mm in diameter, limiting the need for mechanical dissociation. The tissue was placed in 40-50 mm^3^ of cold saline and mixed. Visible blood clots were removed, and the sample was transferred to a 70 µm filter for repeat saline washes with constant stirring. The adipose tissue was then placed in a gentleMACS**™** Dissociator (Miltenyi Biotec) followed by incubation with collagenase D (2 mg/mL). The SVF was separated using a Ficoll-Paque Plus density gradient. Samples were cryopreserved in fetal bovine serum with 10% DMSO in liquid nitrogen.

### 2.3 Library preparation and sequencing

A reagents list can be found in the **Supplementary Data**. An antibody Total Seq C master mix containing 45 common markers for lineage, memory, and activation was created by adding 0.5 µL of each antibody. Twelve samples, with representative samples from the four metabolic groups (non-diabetic PWH, prediabetic and diabetic PWH, and HIV-negative diabetics), were processed at a single time. The samples were quickly thawed and transferred to labeled 15 mL tubes and diluted to 10 ml with phosphate-buffered saline (PBS) before spinning down at 300 G for 10 minutes. The supernatant was aspirated, and the pellet was resuspended in cell staining buffer and transferred to labeled flow tubes. The sample was again spun at 300 G for 10 minutes, and the supernatant was aspirated. Cells were resuspended in 100 µL of staining buffer. Human TruStain FcX Receptor Blocking Solution (5 µL) was added to the tube and incubated at 4 Celsius for 10 minutes. The antibody master mix was spun at 15000 rpm for 5 minutes to remove any aggregates that had formed, and 22.5 µL of the antibody mix was added to each tube of cells and mixed by flicking the tube. A total of one µL of Total Seq C hashtag antibody was added to the samples, and the samples were incubated at 4 Celsius for 30 minutes. After incubation, the cells were washed three times with Cell Staining Buffer and spun at 300 G for 10 minutes. The cells were resuspended in around 100 µL of PBS with 0.04% bovine serum albumin (BSA). The cells were counted using the Countess II Automated Cell Counter (Thermo Fisher) to determine the suspension volume to transfer to obtain 5,000 cells. Four samples (each with a unique hashtag antibody) were pooled by metabolic status (except non-diabetic and prediabetic samples from PWH which were pooled) together into one tube. The multiplexed single cells were loaded onto a Chromium Single Cell 5’ assay (10x Genomics). Libraries were sequenced on the NovaSeq 6000 S2 platform (Illumina). Illumina bcl files were demultiplexed using bcl2fastq. Raw reads were then aligned to the human genome (hg38) using STAR v. 2.7.2a, and cells were called using Cellranger count (v6.0.0) with default settings. Souporcell, which leverages single nucleotide variants to assign individual cells to genotypes and generate a VCF file, was used to genetically demultiplex the samples (27). SoupX was used with default parameters to remove ambient RNA contamination from the count matrices (28).

### 2.4 Quality control

The R Statistical Programming package Seurat V4 was used to further process the scRNA-seq data (29). First, cells with > 25% mitochondrial gene expression, < 800 transcript reads, and < 200 genes were filtered out. The threshold of 800 transcript reads per cell was selected because the performance of Souporcell begins to decrease with fewer transcripts (27). Hashtag oligonucleotides (HTOs) were normalized using centered log-ratio (CLR) transformation, and cells were assigned HTO implemented in the function HTODemux with default parameters (30, 31). We then added the Souporcell assignment to the metadata and linked the Souporcell cluster designation with the HTO classification, forming a link with the metadata. All cells that were unassigned by Souporcell were removed. To identify heterotypic doublets, we used standard processing of each lane, including normalization and variance stabilization using regularized negative binomial regression (SCTransform), dimensional reduction, and clustering (32). DoubletFinder (33), was used to identify potential heterotypic doublets in the data using Souporcell designation as the ground truth. Cells that were identified as doublets by Souporcell or DoubletFinder, clusters where > 60% of cells were identified as doublets, and clusters that expressed transcripts from multiple major lineages (myeloid, lymphoid, stromal, vascular), were removed after integrating the datasets.

### 2.5 Cell clustering and annotation

Processed individual lanes were merged using the merge function. The gene counts were normalized for each cell by dividing by the total gene counts and multiplying by a factor of 10,000 before applying log transformation as implemented by the NormalizeData function. Protein expression counts were normalized using the CLR method. Variable genes were identified using the FindVariableFeatures function (nFeatures = 3000), and the data were scaled and centered using the ScaleData function. Principal component analysis (PCA) was performed on the scaled data. The number of principal components (PCs) used for downstream analysis was selected based on elbow plots and heat maps of PC dimensions as implemented in the DimHeatmap function. To reduce the batch effect associated with running multiple 10X lanes, we used the Harmony algorithm on the uncorrected PCs implemented as a Seurat wrapper to integrate across lanes (34). To evaluate the effectiveness of integration, we used the SCIB pipeline (35). The overall metric ranking was calculated by the summation of the scaled overall bio-conservation score * 0.6 + batch score * 0.4. We performed clustering on the Harmony-corrected PCs using FindNeighbors and FindClusters functions. Marker genes for each cluster were determined using the Wilcoxon Rank Sum test implemented in FindAllMarkers. The integrated dataset was then subclustered as described below. After processing each subcluster, they were merged again, and the process described above (except normalization) was repeated to obtain a cell atlas. We performed manual annotation of cell populations based on canonical markers and markers previously identified in scRNA-seq (**Supplementary Table 4**) (10, 23, 24, 36–49). To compare the annotations of the current dataset with cell annotations from others, we obtained a list of cluster-specific gene markers from prior scRNA-seq datasets for macrophages and stromal cells. We then used these as input into the AddModuleScore function implemented in Seurat to calculate the average expression level for the previously identified cluster-specific markers, which was then scaled. Each cell was labeled with the annotation with the highest module score and overlaid on the UMAP. We also performed SAT cytometric analysis on a subset of individuals in the cohort gating on CD4^+^ CD69^+^ T cells. The methodology was previously described in detail (19).

### 2.6 Subclustering analysis

We subclustered on major cell types, including stromal (*COL1A2, CCDC80*), vascular (*CLDN5, ACTA2*), myeloid (*LYZ, CD68, CD14, CD1C, LILRA4, CLEC9A*), and lymphoid (*CD3, NKG7*). We followed the procedure described previously for each subcluster but did not repeat RNA and protein normalization. Clusters defined by mitochondrial gene expression and/or transcriptional doublets were removed from the analysis. We performed differential gene expression using the FindAllMarkers function implemented in Seurat to identify gene markers for each cell population and included genes that were expressed in 10% or more cells and had a log_2_ fold change of 0.25 or greater.

### 2.7 Composition analysis

Individuals contributing < 30 cells in the subset investigated were excluded. We compared cell composition between disease states for each subcluster by evaluating each cell type as a proportion of total cells in the subcluster. We assessed whether cell type changes were significant between non-diabetic PWH and prediabetic PWH, and between non-diabetic PWH and diabetic PWH using the Wilcoxon Rank Sums test. P values were adjusted for multiple comparisons using Benjamini-Hochberg procedure. We additionally performed differential abundance testing using k-nearest neighbors graphs using MiloR (50). Briefly, the Seurat object was converted to a SingleCellExperiment object and a kNN graph was built using the buildGraph function on the harmony-corrected PCA with k set to 35 and d set to 25. Neighborhoods were defined using the makeNhoods function with random sampling of 10% of cells. Finally, differential abundance testing was performed comparing non-diabetic PWH vs glucose intolerant PWH (prediabetic and diabetic).

To evaluate the independent relationship of cell proportions with BMI, age, and measures of glucose intolerance (HbA1c, FBG), we used partial Spearman’s correlation implemented in PResiduals (51). We used the same method to evaluate the relationship of immune cell proportions with changes in stromal composition. To evaluate the independent relationship of female sex with cell proportions, we used an ordinal linear regression with cell proportion as the outcome and sex (male reference) as the independent variable adjusted for age, BMI, and diabetes status. The β coefficient was converted to an odds ratio (female: male) going from the 25^th^ to 75^th^ percentile (proportion).

### 2.8 Transcriptional analysis

To evaluate differentially expressed genes between non-diabetic and prediabetic PWH, we aggregated gene counts (psuedobulk) for each individual using Scuttle (52). We then used a negative binomial generalized linear model implemented in DESeq2 to evaluate differentially expressed genes adjusting for age, sex, and BMI. We used clusterProfiler to perform gene set enrichment analysis using the Kyoto Encyclopedia of Genes and Genomes (KEGG) and Gene Ontology (GO) biological processes to identify pathways that were enriched in prediabetic compared with non-diabetic PWH (53).

### 2.9 Pseudotime analysis

The R package Slingshot (54), was used to assess the pseudo-time trajectory of PVMs and LAMs. Monocyte-macrophage 2 was specified as the root cluster and end cluster as either PVM or LAM with the dimensionality reduction produced by UMAP. To identify temporally dynamic genes, we fit a generalized additive model (GAM) using a negative binomial additive model implemented in the R package tradeSeq (55). We used the associationTest function with l2fc set to two to identify genes significantly changing along the pseudo-time, defined as FDR-adjusted p-value < 0.05. The genes were then ordered according to pseudo-time and plotted by scaled expression using ComplexHeatmap (56).

### 2.10 Multicellular gene expression programs

To evaluate for a coordinated cellular program that characterizes glucose intolerance, we used DIALOGUE (57), evaluating the cell types we previously identified as associated with glucose intolerance including CD4^+^ and CD8^+^ T cells, macrophage subsets, preadipocytes, and fibroblast cells. DIALOGUE was run with default settings. The multi-level models were fit by glucose intolerance status and adjusted for technical variability, sex, age, and BMI. Average scaled expression of top genes from multicellular program 1 were sorted by expression and samples were plotted with hierarchical clustering (rows) and labeled with clinical variables including BMI, age, sex, and measures of glucose intolerance using ComplexHeatmap.

### 2.11 Intercellular communication analysis

For intercellular ligand-ligand receptor analysis, we used CellChat as implemented in R (58). We compared non-diabetic PWH and glucose intolerant PWH by running CellChat separately on each dataset. Communication probabilities were calculated with population.size set to true and only included genes with expression in 10% or greater of cells in the dataset. Predicted communications were retained in only those with a minimum of ten cells. We then compared the signaling patterns of non-diabetic and glucose intolerant PWH according to the CellChat tutorial. We repeated this analysis to compare the signaling patterns between diabetic HIV-negative persons and diabetic PWH.

### 2.12 Whole adipose tissue messenger RNA expression

The detailed methods for these samples have been published elsewhere (59). Briefly, messenger RNA (mRNA) was extracted from cryopreserved SAT with Qiagen Rneasy Lipid Tissue Kit after mechanical lysis. The NanoString nCounter Plex platform (NanoString, Seattle, WA) was used to quantify mRNA transcripts for 77 genes related to adipocyte function. The mRNA count was normalized using eight synthetic spike-ins for negative control and six synthetic spike-ins for positive controls. The coefficient of variation (CV) was calculated for control genes. First, the mean of the negative controls was used as the background level and subtracted from each gene count. The normalization factor for the mRNA content was calculated using the geometric mean of a set of pre-specified housekeeping genes. The count data were then divided by the normalization factor to generate counts normalized to the geometric mean of housekeeping genes. The normalized gene count data was then log2-transformed, and a linear regression model was used to assess the relationship between the cell-type proportion with mRNA expression. The cell proportion was the independent variable, and the log2-transformed mRNA count was the dependent variable, adjusted for age, sex, BMI, metabolic status, and batch. Benjamini-Hochberg was used to correct for multiple comparisons.

## 3 Results

### 3.1 Cellular composition reflects the complex function of subcutaneous adipose tissue

**Figure 1:**
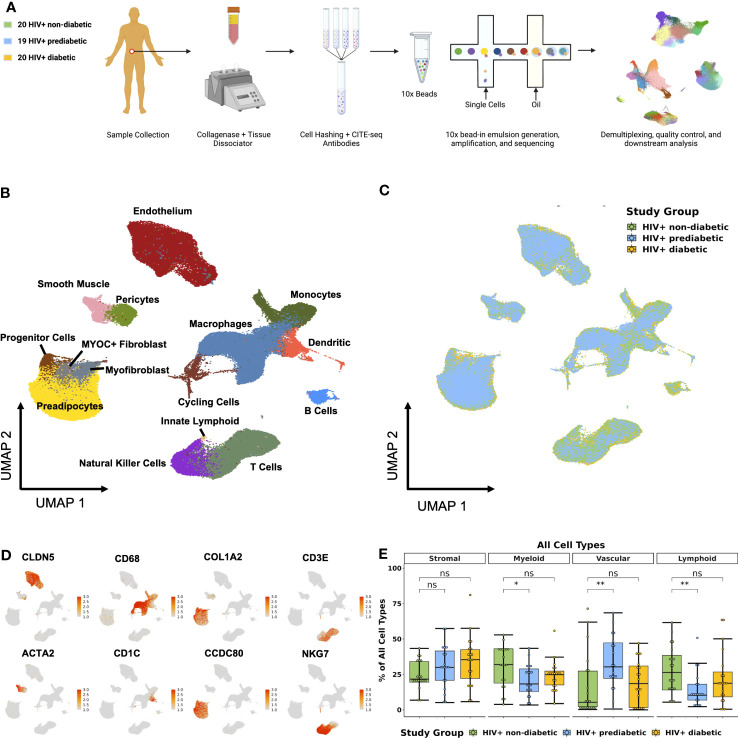
Single-cell RNA sequencing reveals complex cellular composition of subcutaneous white adipose tissue. **(A)** Schematic overview of study design. Twenty HIV+ non-diabetic, 19 HIV+ prediabetic, and 20 HIV+ diabetic participants underwent abdominal subcutaneous adipose tissue harvesting with liposuction. Tissue was processed with collagenase and dissociated. Single-cell suspensions from each participant were hashed and labeled with CITE-seq antibodies before multiplexing in groups of four. 10x libraries were generated using the Chromium platform and sequenced on the Illumina NovaSeq 6000. The bioinformatic pipeline included demultiplexing, quality control, dimensional reduction and clustering, and transcriptional analysis. **(B)** Uniform Manifold Approximation and Projection (UMAP) of 162,552 cells from 59 individuals after removal of doublets and quality control, with manual annotation of cell clusters based on canonical gene markers. **(C)** UMAP after harmony integration grouped by disease status showing successful integration; HIV+ non-diabetic (green), HIV+ prediabetic (blue), and HIV+ diabetic (yellow). **(D)** Gene expression projected onto the UMAP identifying major cell types including stromal (*COL1A2, CCDC80*), vascular (*CLDN5, ACTA2*), myeloid (CD68, CD1C), and T cell and natural killer cells (*CD3E, NKG7*). **(E)** Boxplot showing the proportion of major cell categories (stromal, vascular, lymphoid, and myeloid) as a percentage of total cells split by disease status (n = 59) (HIV+ non-diabetic, green; HIV+ prediabetic, blue; HIV+ diabetic, yellow). The horizontal black line represents the median, the box shows the lower and upper quartile limits and the whiskers are 1.5x the interquartile range. * p < 0.05, ** p < 0.01; ns, not significant.

We enrolled individuals across a spectrum of metabolic health to investigate the role of adipose tissue immune cells in the development of metabolic disease among PWH registered at ClinicalTrials.gov (NCT04451980) (**Table 1**). To comprehensively determine the cellular composition of SAT in the context of glucose intolerance and HIV infection, we collected abdominal SAT from non-diabetic (n = 20), prediabetic (n = 19), and diabetic PWH (n = 20). We used the droplet-based 10X Genomics platform with cellular indexing of transcriptomes and epitopes by sequencing (CITE-seq) (30), to analyze surface phenotypes and transcriptomes (**Figure 1A**; **Supplementary Figure 1A**, methods). After quality control filtering and harmony batch correction (34), (**Supplementary Figures 1B–E**; **Supplementary Tables 1–3**), we obtained a final dataset with 162,552 cells from 59 participants (**Figures 1B, C**). Broadly, cells clustered into stromal, lymphoid, vascular, and myeloid clusters (**Figure 1D**). Cells were annotated using genes previously identified in single-cell datasets (**Supplementary Figure 1F**; **Supplementary Tables 4**, **5**) (15, 40, 42, 44, 47). Importantly, we identified several immune cell types that have been associated with diabetes and obesity in prior studies, including lipid-associated macrophages (LAMs) (40, 60), natural killer (NK) cells (61), gamma delta (γδ) T cells (62), and innate lymphoid cells (ILCs) (15, 63). Prediabetic participants had a greater proportion of vascular cells (p_adj_ = 0.03) and reduced proportion of lymphoid cells (p_adj_ = 0.03), as well as a trend towards a reduced proportion of myeloid cells (p_adj_ = 0.07) compared with non-diabetic participants (**Figure 1E**).

**Table 1:** Cohort characteristics.

	HIV+ non-diabetic (n = 20)	HIV+ prediabetic (n = 19)	HIV+ diabetic (n = 20)	P value
Age, years	46 (40, 52)	42 (38, 54)	55 (48, 62)	0.05
Race, Black (%)	7 (35)	8 (42)	23 (39)	0.99
Sex, female	7 (35)	4 (21)	5 (25)	0.60
Body mass index (kg/m^2^)	31.0 (28.6, 34.7)	29.4 (28.4, 34.2)	33.2 (30.2, 37.8)	0.08
Fasting blood glucose (mg/dl)	88 (82, 92)	111 (102, 122)	184 (120, 242)	< 0.001
Hemoglobin A1c (%)	5.3 (5.1, 5.4)	5.5 (5.1, 5.9)	7.8 (6.4, 9.5)	< 0.001
Metformin use (%)	0 (0)	0 (0)	12 (60)	< 0.001
CD4 count at enrollment	784 (660, 941)	684 (544, 1000)	893 (724, 1102)	0.53
Thymidine analogue exposure (%)	5 (26)	2 (11)	4 (22)	0.45
Integrase-based regimen (%)	13 (65)	11 (58)	11 (55)	0.80

Continuous variables are shown as median value with interquartile range. Categorical variables are shown as number and percent.

**Figure 2:**
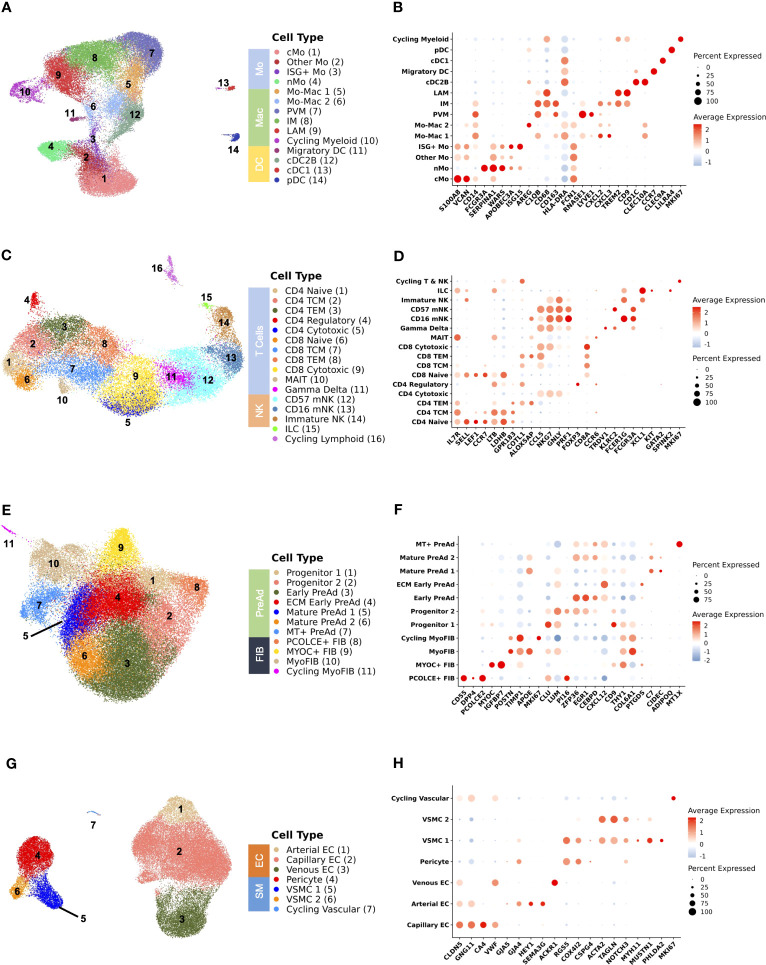
Analysis of subsets shows delineation of cell types that are important for adipose tissue function. **(A)** Uniform Manifold Approximation and Projection (UMAP) of myeloid cells (n = 39,990 cells) from 59 individuals after subsetting and reclustering showing 14 distinct cell types/states. **(B)** Dot plot showing selected myeloid gene markers on the x-axis and cell type on the y-axis. The dot size reflects the percentage of cells expressing the gene while the color reflects the scaled average expression. **(C)** UMAP of T cells, natural killer cells, and innate lymphoid cells (n = 28,061 cells) from 59 individuals after subsetting and reclustering showing 16 distinct cell types/states. **(D)** Dot plot showing selected lymphoid gene markers on the x-axis and cell type on the y-axis. The dot size reflects the percentage of cells expressing the gene while the color reflects the scaled average expression. **(E)** UMAP of stromal cells (n = 50,492 cells) from 59 individuals after subsetting and reclustering. **(F)** Dot plot showing selected stromal gene markers on the x-axis and cell type on the y-axis. The dot size reflects the percentage of cells expressing the gene while the color reflects the scaled average expression. **(G)** UMAP of vascular cells (n = 41,958 cells) from 59 individuals after subsetting and reclustering. **(H)** Dot plot showing selected vascular gene markers on the x-axis and cell type on the y-axis. The dot size reflects the percentage of cells expressing the gene while the color reflects the scaled average expression. cMo, classical monocyte; cDC1, conventional dendritic cell type 1; cDC2B, conventional dendritic cell type 2B; DC, dendritic cell; EC, endothelial cell; ECM, extracellular matrix; FIB, fibroblast; ILC, innate lymphoid cell; IM, intermediate macrophage; ISG+, interferon-stimulated gene +; LAM, lipid-associated macrophage; Mac, macrophage; mNK, mature natural killer; Mo, monocyte; MT, metallothionein+; myoFIB, myofibroblast; NK, natural killer; nMo, non-classical monocyte; pDC, plasmacytoid dendritic cell; PreAd, preadipocyte; PVM, perivascular macrophage; TCM, T central memory; TEM, T effector memory; VSMC, vascular smooth muscle.

We next subset on major cell populations for finer cell annotations. Adipose tissue macrophages and conventional dendritic cells (cDC) interact with adipocytes and can modulate adipogenesis, insulin sensitivity, and tissue remodeling in the setting of obesity (40, 64–67), though there are few similar data in PWH (68, 69). We identified 14 distinct cell populations from 39,990 cells in the myeloid compartment (**Figure 2A**) using canonical gene markers (**Figure 2B**; **Supplementary Table 6**). Surface-marker phenotyping by CITE-seq supported our classification of LAMs (CD11C^+^), perivascular macrophages (PVMs) or M2-like macrophages (CD11C^-^), non-classical monocytes (nMo) (CD16^+^), classical monocytes (cMo) (CD14^+^), and DCs (CD1C^+^) (**Supplementary Figure 2A**). To better characterize the macrophage compartment (**Supplementary Figure 2B**), we performed additional CITE-seq, including several macrophage surface markers, and integrated this with our macrophage dataset. We demonstrate that PVMs expressed *LYVE1* and are CD206^+^CD163^+^CD9^-^, markers associated with the M2 phenotype, while LAMs express *SPP1* and *TREM2* and are CD9^+^ but express significantly lower levels of CD206 and CD163 (**Supplementary Figure 2C**). A subset of macrophages was labeled as intermediate macrophage (IM) and expressed *APOE, APOC1*, and *GPNMB* but had lower expression of *SPP1* and were analogous to a population previously described in mice (**Supplementary Figure 2B**; **Supplementary Table 7**) (40). IM macrophages expressed intermediate levels of CD9 and CD163, and were CD206 negative though gene expression of *MRC1* was detectable at lower levels than in PVM (**Supplementary Figure 2C**). Over-representation analysis demonstrates distinct pathways highlighting functional differences between macrophage subsets (**Supplementary Figure 2D**). We additionally compared our macrophage clusters with previously reported populations from scRNA-seq data (**Supplementary Figures 2E–H**) which show considerable overlap in PVMs and LAMs across all datasets (14, 15, 23, 70). Several precursor populations that we call Mo-Mac 1 and Mo-Mac 2 are grouped within the main cluster identities in other smaller datasets.

Lymphoid cells also have a prominent role in shaping the immune environment of SAT and modulating local inflammation and insulin resistance (13, 71–74). HIV infection induces broad changes to circulating lymphoid cells associated with the development of insulin resistance including memory, senescent, and exhausted phenotypes (19, 21, 22, 75). From 28,061 lymphoid cells, we identified 16 distinct cell states in adipose tissue including CD4^+^ & CD8^+^ naïve (*SELL, LEF1, CCR7*), central memory (T_CM_) (*LTB, LDHB, GPR183*), effector memory (T_EM_) (*ALOX5AP, COTL1, CCL5*), cytotoxic (*NKG7, GNLY, PRF1*), and CD4^+^ regulatory (*FOXP3, CTLA4*) cells. Additionally, we identified γδ T cells (*TRDV1, KLRC2*), mucosal associated invariant T (MAIT) cells (*CCR6, IL7R, KLRG1*), and ILCs *(XCL1, KIT, IL7R*). Several NK cell subsets were identifiable by cell-surface expression of CD16 and CD56 and included mature CD16^+^ (*FCER1G, NKG7)*, terminally differentiated mature CD16^+^CD57^+^ (*FCGR3A, KLRD1*), and CD56^+^CD16^-^ (*XCL1, XCL2, SELL*) (**Figures 2C, D**; **Supplementary Table 6**). The RNA transcriptome profiles were analyzed in parallel with CITE-seq surface marker expression of CD4, CD8, CD45RA, CD27, CD57, and CD16 (**Supplementary Figure 3A**). We also separately assessed only CD4^+^ and CD8^+^ T cells to differentiate more finely between naïve, central memory, effector memory, and cytotoxic phenotypes (**Supplementary Figures 3B–E**; **Supplementary Table 7**). Cells classified as CD4^+^ T_EM_ expressed higher levels of CD69, a marker of tissue residency (**Supplementary Figure 3C**) (76). We validated this finding by performing flow cytometry using SAT single cell suspensions from 21 participants in the same cohort. We observed that the frequency of CD4^+^ CD69^+^ T cells identified by flow cytometry was closely correlated with the proportion of CD4^+^ T_EM_ defined by scRNA-seq (ρ = 0.71, p < 0.001) (**Supplementary Figure 3F**). These data suggest that expression of CD69 is a major indicator of gene expression pattern in SAT T cells.

We next evaluated stromal cell populations, as recent studies have revealed considerable heterogeneity (14, 23, 24, 77–80) and interactions between stromal and immune cells can modulate adipocyte function and have an important role in the development of metabolic disease (67, 81). We identified 11 distinct cells states from 50,492 stromal cells (**Figures 2E, F**; **Supplementary Table 6**). An interstitial fibroblast population (PCOLCE^+^ fibroblast) that has been shown to give rise to preadipocytes expressing *SFRP2, DPP4*, and *PCOLCE2* is analogous to PCOLCE^+^ fibroblasts in the dermis (43), and homologous to a DPP4^+^ population in mice (42). A second fibroblast population has high expression of *MYOC* and *IGFBP7*, which is consistent with anti-adipogenic CD142^+^ cells that have been previously described (77). A separate population of cells expressing *TIMP1* and *POSTN* is transcriptionally consistent with myofibroblasts. Adipose progenitor cells (PCs) were characterized by expression of *DCN*, *CLU, LUM, GSN*, and *PI16*. The preadipocyte compartment was largely differentiated by expression immediate early genes (*MYC, FOS, JUN*) and markers associated with adipogenesis and regulation of inflammation (*ZFP36, EGR1, KLF4, CEBPD*) (Early preadipocyte), and markers associated with extracellular matrix (ECM) (ECM-Producing early preadipocytes). Preadipocytes progressively acquired higher expression of *FABP4, LPL*, and *CIDEC*, which are markers of lipid acquisition. We compared our stromal cell dataset with annotations from other scRNA-seq datasets, which largely shows consistency in the annotation of progenitor/fibro-inflammatory cells, CD142 (anti-adipogenic), and preadipocytes (**Supplementary Figure 3G**) (14, 24, 42, 77, 82). Finally, we found different vascular stromal and endothelial cells present in adipose tissue (**Figures 2G, H**). In summary, we show a diversity of cell types present in SAT in persons with HIV that reflect the complex physiologic functions of adipose tissue.

### 3.2 Categorical and continuous measures of glucose intolerance are associated with macrophage and T cell polarization

Cellular compositional changes of SAT with progressive glucose intolerance likely reflect functional changes that either promote or are derived from the disease process. Several studies evaluating obesity have shown significant changes to the myeloid compartment with an increasing proportion of LAMs and changes to T cell polarization. Less is known about compositional changes to the stromal compartment, though it is known that adipose tissue fibrosis occurs in the setting of obesity (83).

**Figure 3:**
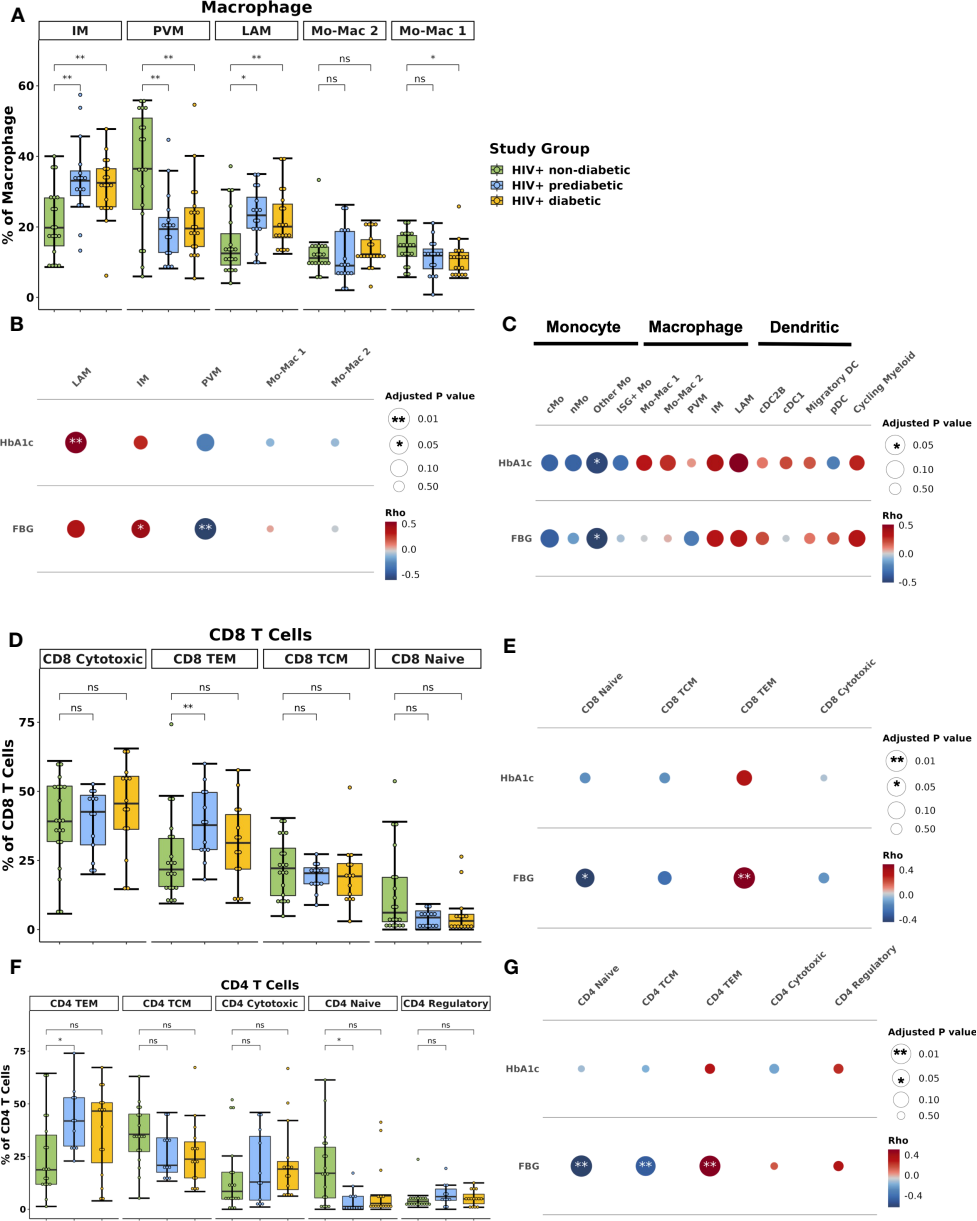
Lipid-associated, intermediate macrophages, and CD4^+^ and CD8^+^ T effector memory proportions are associated with glucose intolerance. **(A)** Boxplot showing the proportion of macrophage types split by disease state (HIV+ non-diabetic, green; HIV+ prediabetic, blue; HIV+ diabetic, yellow) (n = 54). The horizontal black line represents the median, the box shows the lower and upper quartile limits and the whiskers are 1.5x the interquartile range. * p < 0.05, ** p < 0.01; ns, not significant. **(B)** Partial spearman’s correlations between macrophage cell proportions (x-axis) and fasting blood glucose (FBG) or hemoglobin A1c (HbA1c) (y-axis). The area of the circle represents the adjusted p value (larger area = more significant adjusted p-value). Spearman’s ρ is colored red (positive) or blue (negative). **(C)** Partial spearman’s correlations between myeloid cell proportions (x-axis) and FBG or HbA1c (y-axis). **(D)** Boxplot showing the proportion of CD8^+^ T cell subsets as a proportion of total CD8^+^ T cells split by disease state (n = 47). **(E)** Partial spearman’s correlations between CD8^+^ T cell proportions (x-axis) and FBG or HbA1c (y-axis). **(F)** Boxplot showing the proportion of CD4^+^ T cell subsets as a proportion of total CD4^+^ T cells split by disease state (n = 44). **(G)** Partial spearman’s correlations between CD4^+^ T cell proportions (x-axis) and FBG or HbA1c (y-axis). cDC1, conventional dendritic cell type 1; cDC2B, conventional dendritic cell type 2B, cMo, classical monocyte; IM, intermediate macrophage; ISG+ Mo, ISG+ monocyte; LAM, lipid-associated macrophage; Migratory DC, migratory dendritic cell; Mo-Mac, monocyte-macrophage; nMo, non-classical monocyte; Other Mo, other monocyte; pDC, plasmacytoid dendritic cell; PVM, perivascular macrophage; TCM, T central memory; TEM, T effector memory.

Within the macrophage compartment, IM and LAM proportions were higher in prediabetic (p_adj_ = 0.02 and p_adj_ = 0.01) and diabetic (p_adj_ = 0.01 and p_adj_ = 0.01) PWH compared with non-diabetic PWH. In contrast, PVM proportion was lower in prediabetic (p_adj_ = 0.01) and diabetic PWH (p_adj_ = 0.01) compared with non-diabetic PWH (**Figure 3A**). Prediabetic and diabetic PWH had lower proportion of classical monocyte (cMo) (p_adj_ = 0.02 & p_adj_ = 0.10, respectively) and other Mo (p_adj_ = 0.02 & p_adj_ = 0.02, respectively). Compared with non-diabetic PWH, cDC1 proportion was increased in diabetic PWH (p_adj_ = 0.03). We also performed differential abundance testing using k-nearest neighbors graphs using MiloR (50), which yielded similar results (**Supplementary Figures 4A**).

A unique strength of this large single-cell dataset is that concurrently obtained extensive metabolic assessment on study participants provides the opportunity to assess the independent contributions of important biological factors to cellular composition. We examined whether the cell types associated with glucose intolerance in group comparisons were associated with glucose intolerance (hemoglobin A1c [hba1c], fasting blood glucose [FBG]) as a continuous measure. We used partial Spearman’s correlation adjusted for BMI, sex, and age, and we excluded diabetic PWH from the analysis given the effects of medication treatment on the endpoints. FBG was associated with IM proportion (ρ = 0.45, p_adj_ = 0.05) and inversely associated with PVM proportion (ρ = -0.61, p_adj_ = 0.008) (**Figure 3B**; **Supplementary Figure 4B**). HbA1c was associated with LAM proportion (ρ = 0.54, p_adj_ = 0.008) (**Supplementary Figure 4C**). Evaluating all myeloid cells, measures of glucose intolerance were inversely associated with other monocyte cell proportions (**Figure 3C**). Thus, there is a general shift from primarily monocytes and PVMs towards a LAM-like phenotype with glucose intolerance, similar to findings in HIV-negative persons (15).

The lymphoid compartment had fewer differences by diabetes status. The proportion of CD8^+^ T_EM_ was lower in non-diabetic PWH compared with prediabetic (p_adj_ = 0.05) but not diabetic PWH (p_adj_ = 0.44) (**Figure 3D**). FBG but not HbA1c was significantly associated with the proportion of CD8^+^ T_EM_ (ρ = 0.49, p_adj_ = 0.008) and inversely associated with CD8^+^ naïve T cells (ρ = -0.44, p_adj_ = 0.04) (**Figure 3E**; **Supplementary Figure 4D**). Similarly, the proportion of CD4^+^ T_EM_ was lower in non-diabetic PWH compared with prediabetic (p_adj_ = 0.05) but not diabetic PWH (**Figure 3F**). This was mainly due to a significant decrease in CD4^+^ naive T cells (p_adj_ = 0.05). As discussed in the previous section, CD4^+^ T_EM_ cells expressed CD69. We confirmed with flow cytometry that participants with glucose intolerance had higher proportion of CD4^+^ CD69^+^ T cells (median percent: non-diabetic (6.4%), prediabetic (37.5%), diabetic (37.7%); p < 0.05 for both), which is consistent with prior studies (**Supplementary Figure 4E**) (19). FBG was significantly associated with CD4^+^ T_EM_ (ρ = 0.51, p_adj_ < 0.001) and inversely associated with CD4^+^ naïve T cells (ρ = -0.63, p_adj_ < 0.001) (**Figure 3G**; **Supplementary Figure 4F**). The proportion of γδ T cell was increased in prediabetic (p = 0.04) but not diabetic PWH (p = 0.54), though was not significant after correction for multiple comparisons (**Supplementary Figure 4G**). In summary, prediabetic, but not treated diabetic PWH, have increased T_EM_ cells compared with non-diabetic PWH.

Finally, we hypothesized that the proportion of fibroblast populations would be increased with glucose intolerance. However, there was no significant associations between the proportion of stromal cells by diabetes status (**Supplementary Figure 5A**) or by continuous measures of glucose intolerance (**Supplementary Figure 5B**). Similarly, there were no significant differences in the proportions of vascular cells by group or with measures of glucose intolerance (**Supplementary Figures 5C, D**). Taken together, we show that in the context of glucose intolerance, PWH have dramatic changes in the myeloid cell compartment with a shift towards a LAM-like phenotype, as well as a shift towards T_EM_ T cells, but no significant difference in the stromal cell composition.

### 3.3 BMI is associated with macrophage polarization while sex is associated with stromal compositional changes

**Figure 4:**
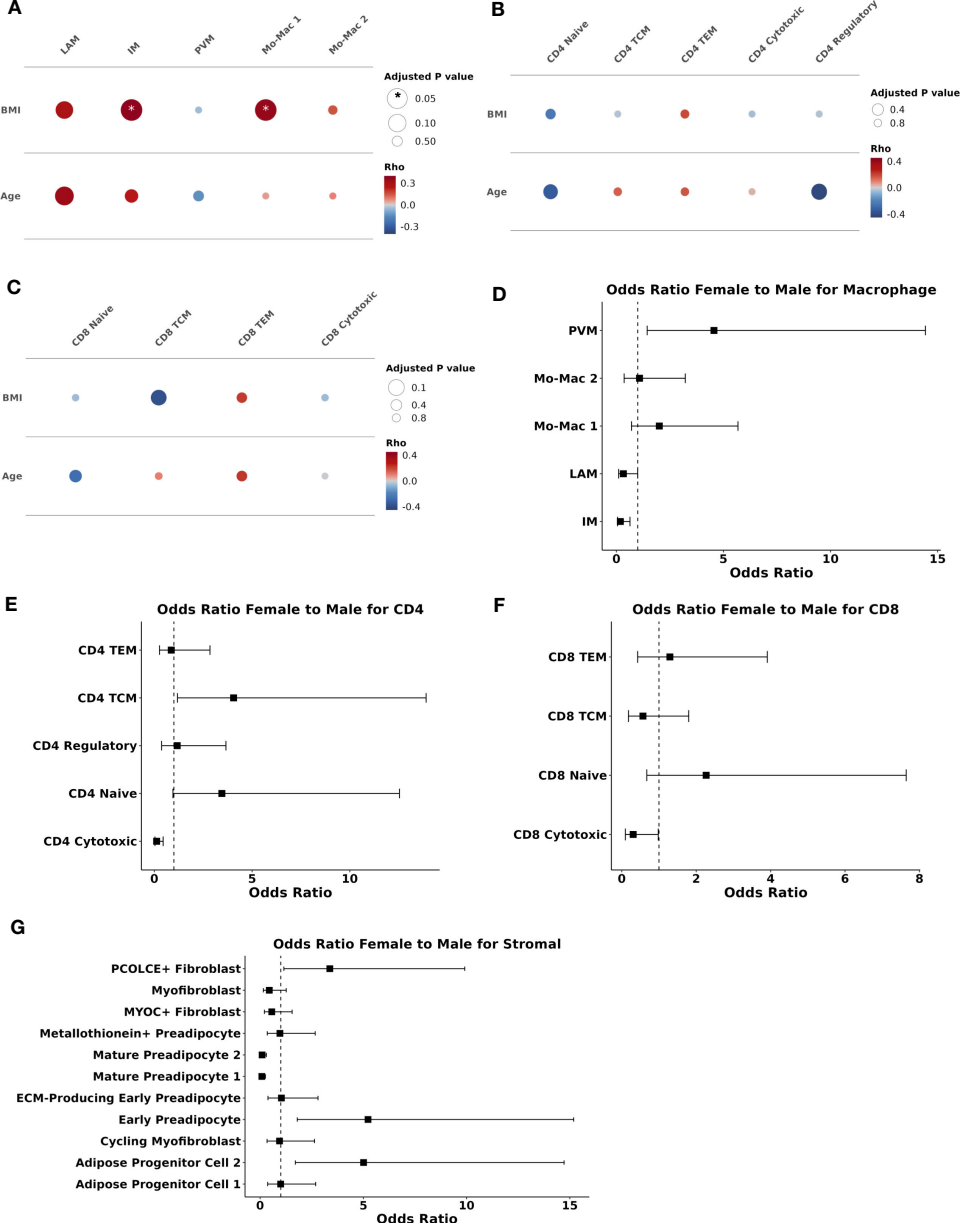
Body mass index and sex are associated with compositional changes in immune and stromal cells. **(A–C)** Partial spearman’s correlations. Spearman’s ρ for the biological factor (body mass index [BMI] or age) and each cluster proportion was calculated. The area of the circle represents the adjusted p value (larger area = more significant adjusted p-value). Spearman’s ρ is colored red (positive) or blue (negative) for **(A)** macrophage, **(B)** CD4^+^ T cells, and **(C)** CD8^+^ T cells. **(D–G)** Ordinal linear regression with cluster proportion as the outcome and sex as the independent variable adjusted for age, BMI, and diabetes status. The regression coefficient for sex was converted into an odds ratio (female:male) and plotted with odds ratio (square) and 95% confidence interval (lines) on the x-axis and cell type on they y-axis for **(D)** macrophage **(E)** CD4^+^ T cells **(F)** CD8^+^ T cells, and **(G)** stromal cells. BMI, body mass index; ECM, extracellular matrix; IM, intermediate macrophage; LAM, lipid-associated macrophage; Mac, macrophage; Mo, monocyte; PVM, perivascular macrophage; TCM, T central memory; TEM, T effector memory.

Demographic variables can influence the adipose tissue environment (10). Therefore, we next examined the relationship of cell composition with BMI, age, and sex. Evaluating macrophages as a percentage of all myeloid cells, BMI was associated with the proportion of IMs (ρ = 0.38, p_adj_ = 0.04) and Mo-Mac 1 (ρ = 0.36, p_adj_ = 0.04) (**Figure 4A**). Older age tended to correlate with a greater proportion of LAMs (ρ = 0.34, p_adj_ = 0.06). In contrast, age and BMI were not significantly associated with changes in proportion of CD4 or CD8 T cell subsets (**Figures 4B, C**). There was a trend towards reduced proportion of CD4 regulatory cells with increasing age (ρ = -0.41, p_adj_ = 0.21). Evaluation of other myeloid, stromal and vascular cell proportions also demonstrated overall weak correlation between BMI or age and cell proportion, except a significant inverse association between BMI and the proportion of capillary endothelial cells (ρ = -0.44, p_adj_ = 0.01) (**Supplementary Figures 6A–C**). In summary, higher BMI and to a lesser extent older age, was associated with a shift towards LAM and LAM-like macrophages and BMI was associated with reduced proportion of capillary endothelial cells.

To assess the independent contributions of sex to SAT composition, we used an ordinal linear regression model adjusted for age, BMI, and diabetes status. Compared with men, women had a higher proportion of PVMs (p_adj_ = 0.02) and lower proportion of IMs (p_adj_ = 0.02), but no significant differences in other myeloid cells (**Figure 4D**). Compared with men, women had a lower proportion of CD4^+^ cytotoxic (p_adj_ = 0.009) but not CD8^+^ cytotoxic T cells (padj = 0.19) (**Figures 4E, F**). In contrast to BMI and age, female sex was significantly associated with stromal cell composition. Compared with men, women had higher proportion of PCOLCE+ fibroblasts (p_adj_ = 0.06), progenitor cell 2 (p_adj_ = 0.009), and early preadipocytes (p_adj_ = 0.009), and lower proportion of mature preadipocytes 1 (p_adj_ < 0.001) and 2 (p_adj_ < 0.001) (**Figure 4G**). Vascular cell proportions were not associated with female sex. Taken together, sex appears to be a larger driver of compositional changes in the stromal compartment than either BMI or age. Female sex is associated with higher proportions of cell populations that are abundant in healthy adipose tissue.

### 3.4 A greater proportion of lipid-associated and intermediate macrophages and CD4^+^ and CD8^+^ T effector memory cells are associated with greater pro-fibrotic cell proportions in stromal tissue

**Figure 5:**
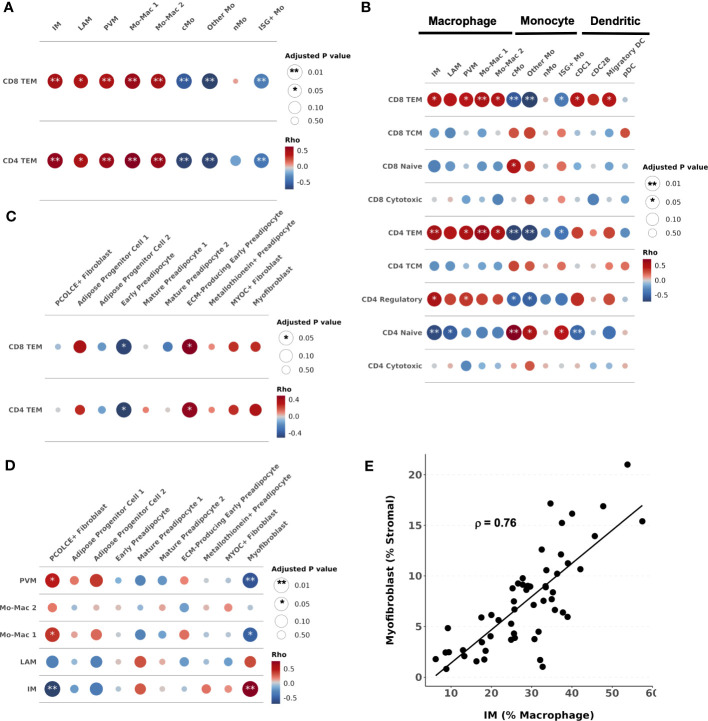
Intercellular correlation of proportions with cells associated with glucose intolerance. **(A–D)** Partial spearman’s correlations. Spearman’s ρ was calculated between the cells of interest (proportion) and each cluster proportion. The area of the circle represents the adjusted p value (larger area = more significant adjusted p-value). Spearman’s ρ is colored red (positive) or blue (negative) for **(A)** CD4^+^ and CD8^+^ T effector memory (T_EM_) proportion and myeloid cell proportions, **(B)** all T cell proportions and myeloid cell proportions, **(C)** CD4^+^ and CD8^+^ T effector memory (T_EM_) proportions and stromal cell proportions, **(D)** and macrophage proportions and stromal cell proportions. **(E)** Scatter plot with intermediate macrophages as a percent of macrophages on the x-axis and myofibroblasts as a percent of stromal cells on the y-axis. cDC1, conventional dendritic cell type 1; cDC2B, conventional dendritic cell type 2B; cMo, classical monocyte; DC, dendritic cell; ECM, extracellular matrix; IM, intermediate macrophage; LAM, lipid-associated macrophage; Mo, monocyte; Mo-Mac, monocyte-macrophage; PVM, perivascular macrophage; pDC, plasmacytoid dendritic cell; TCM, T central memory; TEM, T effector memory.

We next assessed whether a higher proportion of macrophage and T cell phenotypes observed in individuals with glucose intolerance are associated with each other and with changes in the stromal composition. We used partial Spearman’s correlation, adjusted for BMI, age, sex, and diabetes status, and hypothesized that immune cell populations increased with glucose intolerance would be associated with greater pro-fibrotic cell types in adipose tissue. The proportion of CD4^+^ T_EM_ was significantly associated with the proportions of IMs (ρ = 0.57, p_adj_ = 0.001), LAMs (ρ = 0.45, p_adj_ = 0.02), PVMs (ρ = 0.53, p_adj_ = 0.006), and Mo-Mac 1 (ρ = 0.62, p_adj_ < 0.001) and Mo-Mac 2 (ρ = 0.54, p_adj_ = 0.006), and inversely associated with cMo (ρ = -0.70 p_adj_ < 0.001), Other Mo (ρ = -0.70, p_adj_ < 0.001), and ISG+ Mo (ρ = -0.42, p_adj_ = 0.008) (**Figure 5A**). Similarly, the proportion of CD8^+^ T_EM_ was also associated with the proportions of IMs (ρ = 0.48, p_adj_ = 0.006), LAMs (ρ = 0.43, p_adj_ = 0.02), PVMs (ρ = 0.44, p = 0.006), and Mo-Mac 1 (ρ = 0.53, p_adj_ = 0.001) and Mo-Mac 2 (ρ = 0.49, p_adj_ = 0.009), and inversely associated with cMos (ρ = -0.57, p_adj_ = 0.001), Other Mo (ρ = -0.72, p_adj_ < 0.001), and ISG+ Mo (ρ = -0.38, p_adj_ = 0.007) (**Figure 5A**). Additionally, we expanded this analysis to include all T cell subsets that showed naïve CD4^+^ and CD8^+^ T cells were associated with greater monocyte proportions (**Figure 5B**).

The proportion of CD4^+^ T_EM_ was associated with the proportion of ECM-producing early preadipocytes (ρ = 0.46, p_adj_ = 0.03) and inversely associated with early preadipocytes (ρ = -0.51, p_adj_ = 0.03) (**Figure 5C**). The proportion of CD8^+^ T_EM_ was similarly associated with the proportion of ECM-producing early preadipocytes (ρ = 0.49, p_adj_ = 0.03) and inversely associated with early preadipocytes (ρ = -0.49, p_adj_ = 0.03) (**Figure 5C**). The proportion of IMs (% of macrophages) was associated with the proportion of myofibroblasts (ρ = 0.76, p_adj_ < 0.001) and inversely associated with PCOLCE+ fibroblasts (ρ = -0.70, p_adj_ < 0.001) (**Figures 5D, E**). The proportion of PVMs was associated with PCOLCE+ fibroblasts (ρ = 0.46, p_adj_ = 0.02) and inversely associated with myofibroblasts (ρ = -0.57, p_adj_ < 0.001) (**Figure 5D**). The relationship between stromal cells and other T cells or myeloid cells are shown in **Supplementary Figures 6D, E**. Taken together, we find high correlation between the proportion of T_EM_ cells, macrophages, and fibroblast cell populations in SAT, suggesting a coordinated shift in the immune cell and stromal cell lineages accompanies changes in metabolic health.

### 3.5 Glucose intolerant individuals have transcriptional polarization of macrophages towards LAMs and T cells towards effector memory phenotype

**Figure 6:**
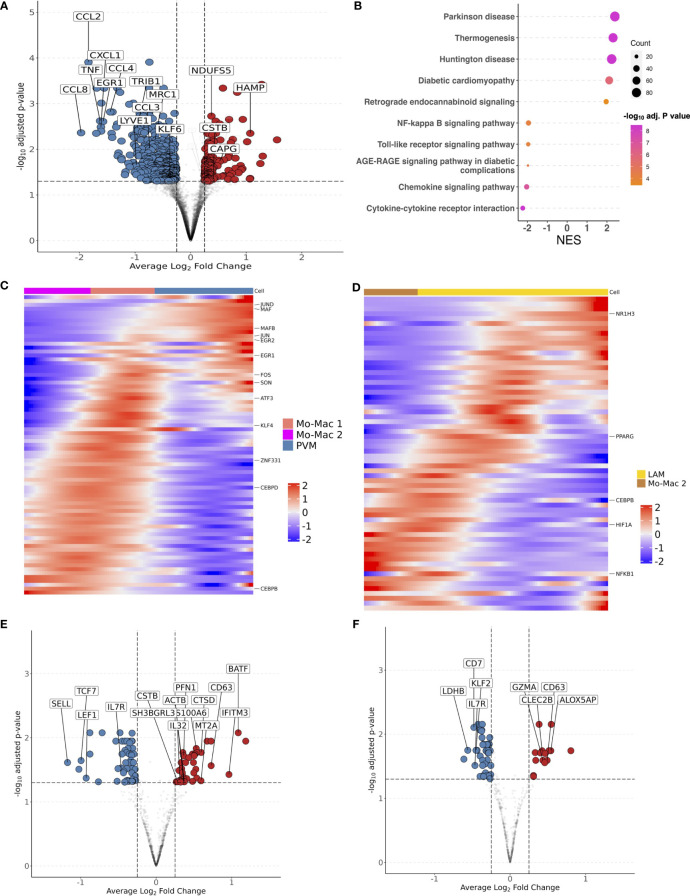
Transcriptional shift from immunoregulatory to metabolic phenotype in macrophages and effector memory phenotype in T cells with glucose intolerance. **(A)** Macrophage volcano plot with average Log_2_ fold change (x-axis) and –log_10_ adjusted p-value (y-axis) for prediabetic vs non-diabetic (reference) PWH. Genes that had ≥ 0.25 log_2_ fold change and adjusted p-value < 0.05 were colored red (higher expression) and blue (lower expression). **(B)** Gene set enrichment analysis (GSEA) using the KEGG database. The top over and under enriched pathways were included with normalized enrichment score (NES) on x-axis and descriptive term on y-axis. Dot size represents the number of gene hits in the pathway and dot color represents the –log_10_ adjusted p-value. **(C)** Ordered and smoothed transcription factor gene expression (scaled) along the pseudotime trajectory for monocyte-macrophage 2 to perivascular macrophage. Selected genes were significantly differentially expressed along the pseudotime with ≥ log_2_(2) fold change according to TradeSeq. **(D)** Ordered and smoothed transcription factor gene expression (scaled) along the pseudotime trajectory for monocyte-macrophage 2 to lipid associated macrophages. Selected genes were significantly differentially expressed along the pseudotime with ≥ log_2_(2) fold change according to TradeSeq. **(E)** CD4^+^ T cell volcano plot with average Log_2_ fold change (x-axis) and –log_10_ adjusted p-value (y-axis) for prediabetic vs non-diabetic (reference) PWH. Genes that had ≥ 0.25 log_2_ fold change and adjusted p-value < 0.05 were colored red (higher expression) and blue (lower expression). **(F)** CD8^+^ T cell volcano plot with average Log_2_ fold change (x-axis) and –log_10_ adjusted p-value (y-axis) for prediabetic vs non-diabetic (reference) PWH. Genes that had ≥ 0.25 log_2_ fold change and adjusted p-value < 0.05 were colored red (higher expression) and blue (lower expression). LAM, lipid-associated macrophage; Mo-Mac, monocyte-macrophage; PVM, perivascular macrophage; NES, normalized enrichment score.

Having demonstrated that glucose intolerance is independently associated with accumulation of IM, LAM, CD4^+^ T_EM_, and CD8^+^ T_EM_, and inversely associated with PVM (or M2-like macrophages), we next examined the transcriptome that defines these cell compartments. We aggregated gene counts for each participant (pseudobulk method) to evaluate the differential gene expression between prediabetic and non-diabetic PWH using a negative binomial generalized linear model implemented in DESeq2 (84), adjusting for age, sex and BMI. 597 genes were differentially expressed (p_adj_ < 0.05) between macrophages from prediabetic and non-diabetic PWH. Macrophages from prediabetics had higher expression of genes related to oxidative phosphorylation (*NDUFS5, NDUFAB1, ATP5MC3, UQCR10, COX5B*), and lower expression of genes related to chemotaxis (*CCL8, CXCL1, CCL2, CCL4L2, CCL4, CCL3L1, CXCL8, CCL3*), TNF inflammatory pathway (*TNF, TNFSF18, TNFRSF1A, TNFRSF21*), and M2 macrophage polarization (*TRIB1, EGR1, EGR2, MRC1, LYVE1, KLF4*) (**Figure 6A**; **Supplementary Table 8**) (85, 86). KEGG gene set enrichment analysis (GSEA) confirmed enrichment of metabolic disease pathways and reduction in pathways related to cytokine-cytokine receptor interaction, chemokine signaling, and toll-like receptor signaling (**Figure 6B**). Individual macrophage subsets had fewer transcriptional differences between non-diabetic and prediabetic PWH. In PVMs, several genes related to chemokines and M2-like macrophages (*CCL2, CCL3, CCL4*) trended towards lower expression in prediabetic PWH (p_adj_ < 0.06) while *ID3* had higher expression (**Supplementary Table 8**). Pseuodotime using Slingshot (54), demonstrated a single trajectory from monocyte-macrophage 2 transitioning into PVMs (**Supplementary Figure 7A**). Using Tradeseq (55), which fits a negative binomial generalized additive model to each gene, expression of transcription factors associated with M2 macrophage phenotype increased along the pseudotime trajectory including *ZNF331, NR4A3, KLF4, KLF2, MAF, MAFB, ATF4, EGR2, CEBPD, JUND*, and *SON* (**Figure 6C**; **Supplementary 7B**). Several of these genes had reduced expression in prediabetic compared with non-diabetic PWH with pseudobulk differential gene expression analysis. Despite their large proportion, IMs had few differentially expressed genes, between prediabetic and non-diabetic PWH (**Supplementary Table 8**). It is not clear based on pseudotime whether these cells are transitional or represent a terminal state of differentiation. Finally, LAMs in prediabetic PWH had higher expression of lipid-processing/metabolic genes (*MTLN*, *COX6A1, NDUFB3*), and lower expression of several M2 macrophage transcription factors (*TRIB1, EGR1, KLF2*) (**Supplementary Table 8**). Pseudotime suggested a transition from monocyte-macrophage precursors to LAMs (**Supplementary Figure 7C**) with upregulation of several transcription factors including *PPARG* (**Figure 6D**; **Supplementary Figure 7D**), which has been shown to influence CD36 expression and have a role in lipid metabolism (87).

CD4^+^ T cells in prediabetic PWH had higher expression of markers of activation and effector memory phenotype (*IL32, BATF, CD63, IFITM3*) and lower expression of markers related to naïve T cells (*SELL, LEF1*) (**Figure 6E**). CD8^+^ T cells in prediabetic PWH had higher expression of cytotoxic and activation genes (*GZMA, CD63, CLEC2B*) (**Figure 6F**).

There were no differentially expressed genes in preadipocytes from prediabetic versus non-diabetic PWH (**Supplementary Table 8**). Comparison of diabetic with non-diabetic PWH showed similar, but fewer differentially expressed genes between immune cells, which could reflect treatment effect (**Supplementary Table 9**). Taken together, macrophages show a transcriptional profile that shifts from an immune regulatory M2-like macrophage to a metabolic phenotype in prediabetic PWH. The expression of transcription factors associated with M2 macrophage polarization is reduced in prediabetic PWH, suggesting decreased differentiation of monocyte-macrophages into PVMs/M2 macrophages. The T cell transcriptional profile shifts towards an activated effector memory/antigen presentation phenotype in prediabetic PWH. While most of the differentially expressed genes were unique by cell type, there were overlapping genes between cell types in both higher and lower expressed genes, particularly between CD4^+^ and CD8^+^ T cells and between macrophage subsets (**Supplementary Figures 7E, F**).

### 3.6 Intercellular gene programs related to interferon-γ, tumor necrosis factor-α, and lipid metabolic processes characterize glucose intolerance

**Figure 7:**
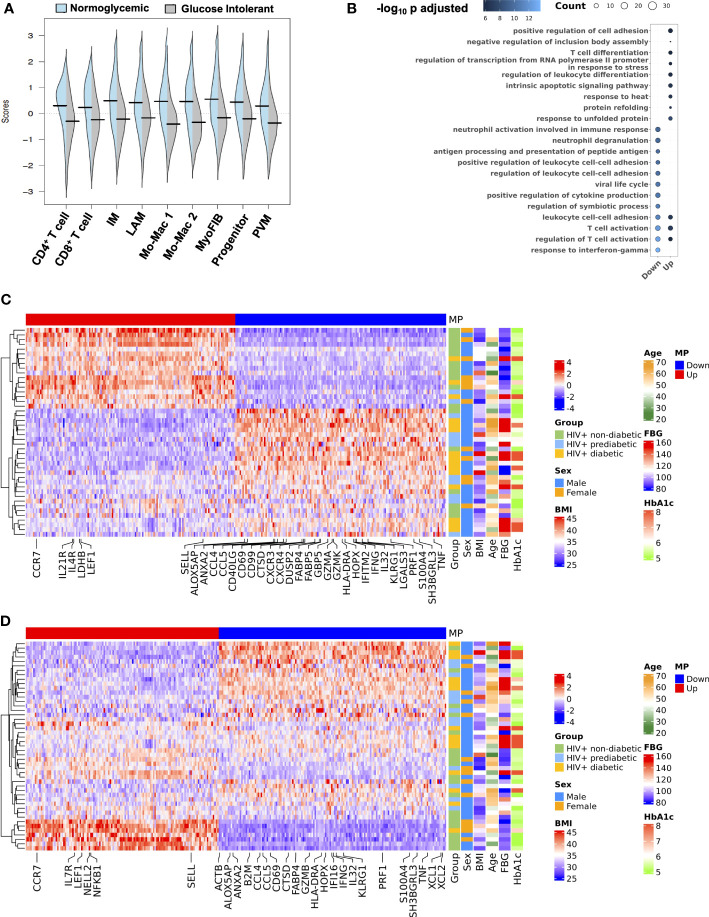
An intercellular gene expression program enriched for interferon-γ, tumor necrosis factor, and lipid metabolism defines glucose intolerance. **(A)** Distribution of expression scores for each cell type component for multicellular program (MP) 1 with kernel density estimates. **(B)** Over-representation analysis using Gene Ontology biological process for genes in the up and down compartments in MP1. Dot size represents the number of gene hits in the pathway and dot color represents the –log_10_ adjusted p-value. **(C)** Average scaled expression of top CD4^+^ T cell genes from MP1 sorted by expression (columns), across samples plotted with hierarchical clustering (rows) and labeled with clinical variables including body mass index (BMI), age, sex, and measures of glucose intolerance. **(D)** Average scaled expression of top CD8^+^ T cell genes from MP1 sorted by expression (columns), across samples plotted with hierarchical clustering (rows) and labeled with clinical variables including BMI, age, sex, and measures of glucose intolerance. BMI, body mass index; FBG, fasting blood glucose; HbA1c, hemoglobin A1c; IM, intermediate macrophage; LAM, lipid-associated macrophage; Mo-Mac, monocyte-macrophage; MP, multicellular program; MyoFIB, myofibroblast; PVM, perivascular macrophage.

Based on some overlapping features of gene changes, and an overall shift in transcriptional profile, we next assessed whether there were coordinated, inter-cellular gene expression programs that define the transcriptional patterns associated with glucose intolerance. We employed DIALOGUE, which is a dimensionality reduction technique that identifies gene expression programs between cell types to identify tissue-specific cellular programs (57). Multicellular program 1 (MP1) was highly associated with normoglycemia while glucose intolerance was inversely related to MP1 (**Figure 7A**). Over-representation analysis of the MP1 showed some overlap, but enrichment of genes related to leukocyte differentiation, T cell differentiation, and protein folding in its upregulated compartment. In contrast, MP1 downregulated compartment was enriched in genes related to IFN-γ responses, neutrophil degranulation, and antigen processing and presentation (**Figure 7B**). Glucose intolerance was highly related to CD4^+^ T cell gene expression pattern (**Figure 7C**). In CD4^+^ T cells, non-diabetic individuals had increased expression of several genes including *CCR7, LDHB, LEF1, SELL, IL21R* and several ribosomal proteins consistent with a less differentiated state, while prediabetic and diabetic individuals had greater expression of genes related to cytotoxicity (*CCL5, CCL4, PRF1*), activation (*IL32, CD40LG, HLA-DRA*), and inflammation (*GBP5, IFNG, TNF, IFITM2)*. Several individuals classified as non-diabetic or prediabetic were borderline based on dichotomous classification, which could explain why some individuals did not cluster as expected. Additionally, several non-diabetic individuals who clustered with glucose-intolerant individuals were cytomegalovirus (CMV) seropositive and had a high proportion of cytotoxic T cells based on flow cytometry, likely in response to CMV infection. This suggests that while glucose intolerance is highly associated with MP1, other factors can contribute to an inflammatory SAT environment. CD8^+^ T cells showed a similar expression pattern; however it did not associate as strongly with glucose intolerance (**Figure 7D**). The macrophage compartment MP1 expression program was also not as strongly associated with diabetes status as the T cell compartment but did show enrichment of genes associated with M2 macrophage polarization (*KLF4, MAFF, MAFB, ATF4, EGR1, EGR2*) and chemotaxis (*CCL2,CXCL8*) in MP1 upregulated compartment, and enrichment of genes associated with lipid metabolism (*TREM2*), IFN-γ (IFI27, ISG15), and macrophage activation (*AREG*) in MP1 downregulated compartment (**Supplementary Figures 8A–C**). Diabetes status was also significantly associated with MP1 in myofibroblast (**Supplementary Figure 8D**). Genes associated with glucose intolerance phenotype included genes related to ECM/cell-cell interaction *(BGN, COL4A1, PRSS23, TNC, TAGLN).* Overall, MP1 differentiates between normoglycemic individuals and glucose intolerant individuals with the tissue program shifting towards genes related to cytotoxicity, inflammation, lipid metabolism, macrophage activation, and ECM deposition (**Supplementary Figure 8E**).

### 3.7 Intercellular communication analysis reveals enrichment of pro-inflammatory and pro-fibrotic pathways with glucose intolerance

**Figure 8:**
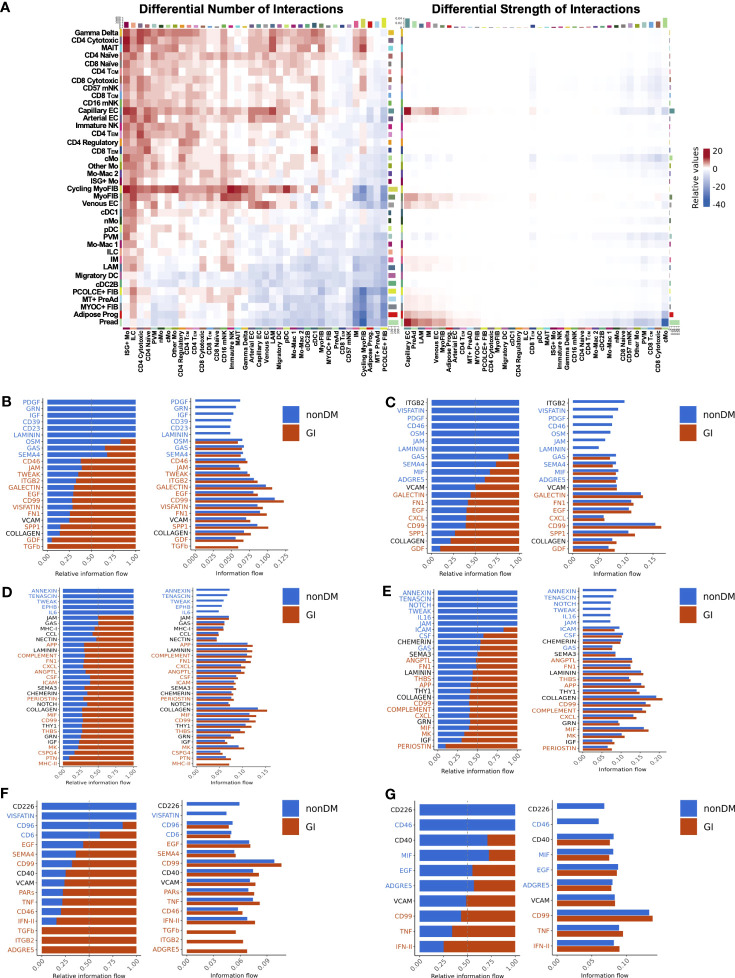
Intercellular communication analysis predicts enhanced signaling of pro-fibrotic, pro-inflammatory pathways in PWH with glucose intolerance (GI). **(A)** Relative number of interactions (left) and strength of interactions (right) comparing PWH with glucose intolerance and non-diabetic (nonDM) PWH. Increased and decreased relative signaling are shown in red and blue, respectively. The target cells are shown on the x-axis and the source cells are shown on the y-axis. Rows and columns were plotted with hierarchical clustering. **(B–G)** Bar plot with relative information flow (left) or overall information flow (right) on the x-axis and predicted ligand-ligand receptor pathways from source cells to target cells. Signaling with glucose intolerance is shown in orange while signaling in non-diabetics is shown in blue. Pathways with significantly greater interaction with glucose intolerance based on Wilcoxon rank sum are labeled in orange while pathways with significantly greater interaction in non-diabetic are labeled in blue (p < 0.05). **(B)** Intermediate macrophage (source) to myofibroblast and cycling myofibroblast (target). **(C)** Intermediate macrophage (source) to preadipocyte and progenitor cells (target) **(D)** Cycling myofibroblast and myofibroblast (source) to intermediate macrophage (target). **(E)** Preadipocyte and progenitor cells (source) to intermediate macrophages (target). **(F)** CD4^+^ T_EM_ (source) to myofibroblast and cycling myofibroblast (target). **(G)** CD4^+^ T_EM_ (source) to preadipocyte and progenitor cells (target). cMo, classical monocyte; cDC1, conventional dendritic cell type 1; cDC2B, conventional dendritic cell type 2B; DC, dendritic cell; EC, endothelial cell; FIB, fibroblast; ILC, innate lymphoid cell; IM, intermediate macrophage; ISG+, interferon-stimulated gene +; LAM, lipid-associated macrophage; Mac, macrophage; mNK, mature natural killer; Mo, monocyte; MT, metallothionein+; myoFIB, myofibroblast; NK, natural killer; nMo, non-classical monocyte; pDC, plasmacytoid dendritic cell; PreAd, preadipocyte; Prog, progenitor; PVM, perivascular macrophage.

Glucose intolerant and non-diabetic PWH have striking differences in tissue level gene expression programs. We next wanted to assess whether intercellular signaling that coordinates cell function and expression programs was different between glucose intolerant and non-diabetic PWH. We employed CellChat to infer intercellular communications and identify differential ligand-ligand receptor interactions between glucose intolerant and non-diabetic PWH (58). Overall, the number of predicted interactions was increased in glucose intolerant individuals from lymphoid, endothelial, myofibroblast, and to a lesser extent myeloid populations (source) to several lymphoid, myeloid, and endothelial cell populations (target) compared with non-diabetic PWH (**Figure 8A**). Individuals with glucose intolerance also had greater interaction strength between cycling myofibroblast, myofibroblast, capillary endothelium, preadipocytes, progenitor cells, and, to a lesser extent, IMs and LAMs with endothelium, preadipocytes, LAMs, IMs, myofibroblasts, and CD4^+^ T_EM_ cells (**Figure 8A**). Given the multitude of potential intercellular communications, we prioritized cell populations we have previously linked with glucose intolerance. IM were predicted to have increased interaction with myofibroblast through TGF-β, growth differentiation factor, osteopontin, visfatin, fibronectin, tweak, and integrin subunit beta 2 pathways in glucose intolerant individuals compared with non-diabetic PWH (**Figure 8B**), which have been implicated in fibrosis and inflammation (88–91). Predicted cell-cell communication between IM and preadipocytes in glucose intolerant individuals also included growth differentiation factor, osteopontin, CD99, CXCL, galectin, and fibronectin pathways (**Figure 8C**). LAMs had similar predicted interactions as IMs (**Supplementary Figures 9A–D**). We also evaluated predicted signaling from myofibroblast (**Figure 8D**) and preadipocytes (**Figure 8E**) to IMs, which showed increased MHC-II, CD99, MIF, periostin, amyloid beta precursor protein (APP), and CSF signaling pathways with glucose intolerance and have been implicated in macrophage polarization (92–94). CD4^+^ T_EM_ were predicted to have a greater interaction through TNF and IFN pathways with myofibroblast and preadipocytes in glucose intolerant individuals compared with non-diabetic individuals (**Figures 8F, G**). Greater interactions through IFN, TNF, CCL, and MIF pathways from T_EM_ cells to IMs and LAMs were predicted for glucose intolerant individuals compared with non-diabetic persons (**Supplementary Figures 9E, F**). Overall, intercellular communication analysis shows an increased number or strength of interactions with myofibroblasts, preadipocytes, lymphoid cells, and IMs and LAMs, with pathways predicted to promote ECM deposition and immune cell polarization in persons with glucose intolerance.

### 3.8 CD4^+^ T_EM_ cells expressing CD69 are associated with changes in mature adipocyte gene expression

CD4^+^ T_EM_ proportion and transcriptional expression were strongly associated with glucose intolerance compared with other immune cell types. These cells express CD69, which is often a marker of tissue residency (76). Given their close association with glucose intolerance and multicellular program 1, we next assessed whether these cells are associated with changes in adipocyte gene expression patterns. We performed probe-based RNA transcript quantification of whole adipose tissue biopsies for 77 adipocyte genes using the NanoString platform. We found that cytometric sorted CD4^+^CD69^+^ T cells and single-cell CD4^+^ T_EM_ cell proportions had similar relationships with adipocyte gene expression with higher expression of *ADIPOQ, LPL*, and *LEP*, and lower expression of genes related to long-chain fatty acid metabolism (*CPT1B, CYP27A1, SLC27A5, ACAA1*) and GLP1R in whole SAT (**Supplementary Table 10**). No other immune cell subsets, including macrophage types or CD8^+^ T_EM_ had a significant relationship with adipocyte gene expression. Taken together, changes in SAT CD4^+^ tissue-resident cell composition are associated with changes in adipocyte gene expression, which suggests a potential mechanistic link between SAT immune cells, mature adipocytes, and development of metabolic phenotypes.

### 3.9 HIV-negative diabetic and HIV-positive diabetic individuals have similar SAT composition and multicellular gene expression programs, but divergent intercellular communication pathways

**Figure 9:**
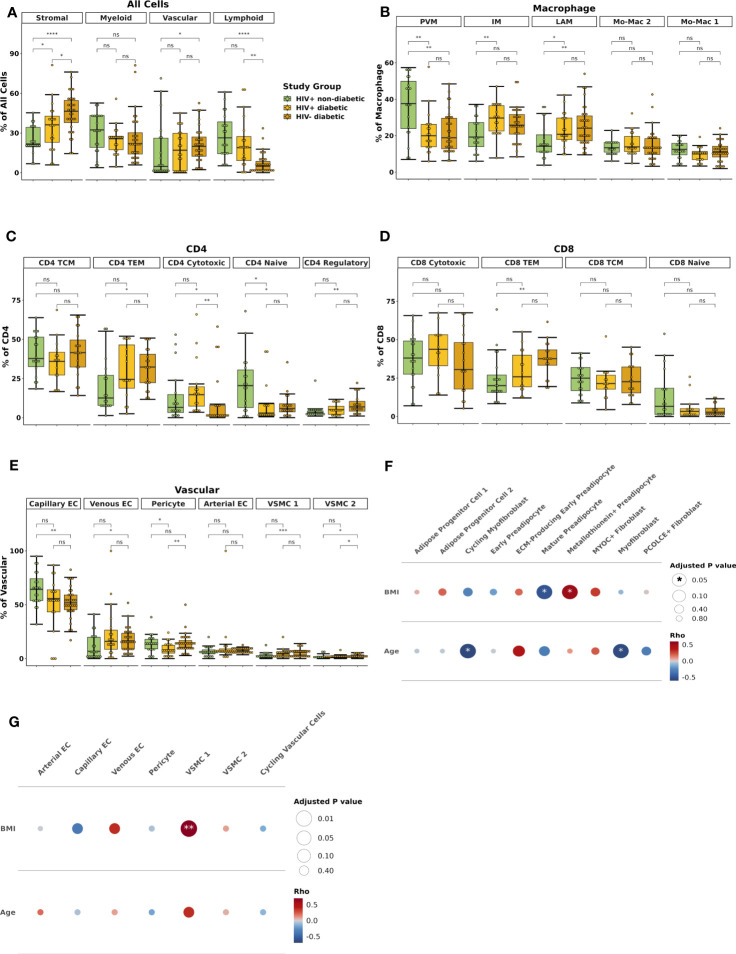
HIV-negative diabetic and HIV-positive diabetic have similar macrophage and T effector memory cell polarization. **(A)** Box plot showing the proportion of major cell categories (stromal, vascular, lymphoid, and myeloid) as a percentage of total cells split by disease state (HIV+ non-diabetic, green; HIV+ diabetic, yellow; HIV- diabetic, orange) (n = 72). The horizontal black line represents the median, the box shows the lower and upper quartile limits and the whiskers are 1.5x the interquartile range. * p < 0.05, ** p < 0.01, *** p < 0.001, **** p < 0.0001; ns, not significant. **(B)** Boxplot showing the proportion of macrophage subsets as a percentage of total macrophage cells split by disease status (n = 70). **(C)** Boxplot showing the proportion of CD4^+^ T cell subsets as a percentage of total CD4^+^ T cells split by disease status (n = 52). **(D)** Boxplot showing the proportion of CD8^+^ T cell subsets as a percentage of total CD8^+^ T cells split by disease status (n = 51). **(E)** Boxplot showing the proportion of vascular subsets as a percentage of total vascular cells split by disease status (n = 71). **(F, G)** Partial spearman’s correlations in HIV-negative diabetic individuals only. Spearman’s ρ for the biological factor (body mass index [BMI] or age) and each cluster proportion was calculated. The area of the circle represents the adjusted p value (larger area = more significant adjusted p-value). Spearman’s ρ is colored red (positive) or blue (negative) for **(F)** stromal and **(G)** vascular cells. BMI, body mass index; EC, endothelial cell; ECM, extracellular matrix; IM, intermediate macrophage; Mo-Mac, monocyte-macrophage; LAM, lipid-associated macrophage; PVM, perivascular macrophage, TCM, T central memory; TEM, T effector memory; VSMC, vascular smooth muscle cell.

Finally, we performed an exploratory analysis to assess whether the regulatory patterns that define glucose intolerance in PWH are similar in diabetic HIV-negative persons. To this end, we recruited a smaller group of 32 HIV-negative persons, all of whom had diabetes, with clinical and demographic features similar to the diabetic PWH (**Supplementary Table 11**). Single cell assays were performed at the same time (**Supplementary Tables 1**, **2**), and in the same manner, as in PWH, and we integrated datasets of these two groups to assess for differences by HIV status. We recovered the same cell types in HIV-negative persons observed in the PWH (**Supplementary Figure 10A**). Compared with the cell distribution of non-diabetic and diabetic PWH, HIV-negative persons had fewer lymphoid (p_adj_ < 0.001 and p_adj_ = 0.006) and greater stromal cells as a proportion of all cells (p_adj_ < 0.001 and p_adj_ = 0.05) (**Figure 9A** **)**. In the macrophage compartment, there were no significant difference in composition between diabetic PWH and diabetic HIV-negative persons (**Figure 9B**). Non-diabetic PWH had more PVMs (p_adj_ = 0.02) and less LAMs (p_adj_ = 0.03), but no difference in IMs (19.3% vs 25.8%) after correction for multiple comparisons. Non-diabetic PWH tended to have fewer CD4^+^ T_EM_ cells (p_adj_ = 0.09) though this was not significant after correction for multiple comparisons (**Figure 9C**). Non-diabetic PWH had fewer CD8^+^ T_EM_ than HIV-negative diabetic persons (p_adj_ = 0.03) but there were no other significant compositional differences (**Figure 9D**). Within the vascular cells, non-diabetic PWH had greater capillary endothelial cells (p_adj_ = 0.05), fewer VSMC 1 (p_adj_ = 0.004), and a trend towards fewer VSMC 2 (p_adj_ = 0.06) (**Figure 9E**). Diabetic PWH had fewer pericytes than diabetic HIV-negative persons (p_adj_ = 0.05). No significant differences were observed in stromal cell composition.

We also examined whether BMI and age influence SAT composition in HIV-negative diabetics. We found, in contrast to PWH, that BMI was significantly correlated with the proportion of metallothionein+ preadipocytes (ρ = 0.51, p_adj_ = 0.02) and inversely associated with the proportion of mature preadipocytes (ρ = -0.49, p_adj_ = 0.02), likely because mature adipocytes express greater metallothionein genes (**Figure 9F**). Age was inversely associated with the proportion of myofibroblasts and cycling myofibroblasts (p_adj_ = 0.02 for both). BMI was also significantly associated with the proportion of VSMC 1 (ρ = 0.69, p_adj_ < 0.001), which has been associated with obesity in previous studies (**Figure 9G**) (95). The relationship of BMI or age with other cell types, including CD4^+^ T cells, CD8^+^ T cells, myeloid, and lymphoid cells, were not significant after correction for multiple comparisons except for BMI and CD4^+^ naive (**Supplementary Figures 10B–E**). In summary, HIV-negative diabetic persons have greater proportion of LAMs and CD8^+^ T_EM_ cells but no difference in IMs compared with non-diabetic PWH. Although lymphoid cells are more prevalent in diabetic PWH, the overall distribution is relatively similar with HIV-negative diabetic individuals.

**Figure 10:**
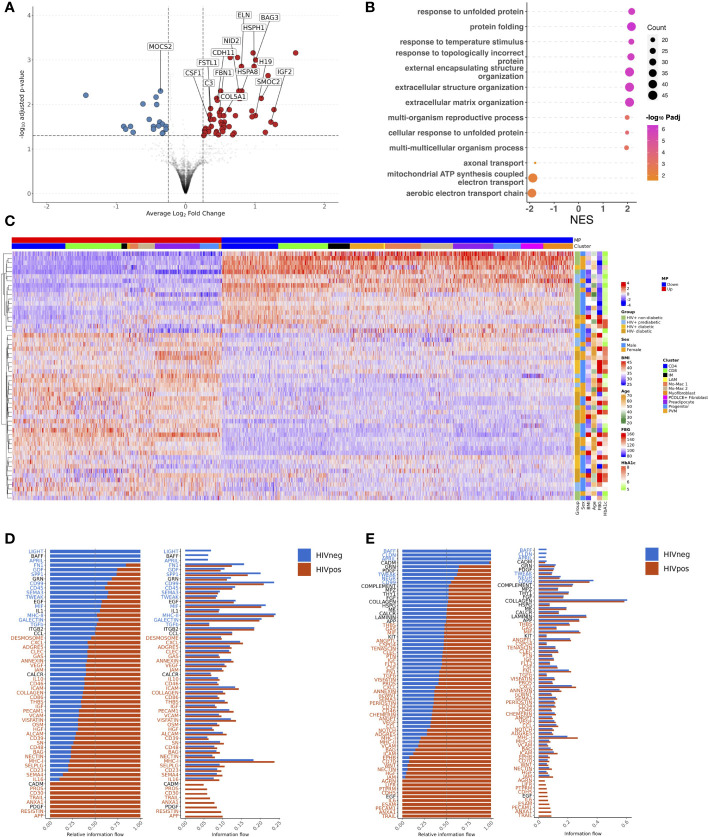
HIV-negative diabetic and HIV-positive diabetic have a similar multicellular gene expression program but divergent intercellular communication pathways. **(A)** Preadipocyte volcano plot with average Log_2_ fold change (x-axis) and –log_10_ adjusted p-value (y-axis) for HIV-positive diabetic vs HIV- diabetic (reference) persons. Genes that had ≥ 0.25 log_2_ fold change and adjusted p-value < 0.05 were colored red (higher expression) and blue (lower expression). **(B)** Gene set enrichment analysis (GSEA) using the Gene Ontology database. The top over and under enriched pathways were included with normalized enrichment score (NES) on x-axis and descriptive term on y-axis. Dot size represents the number of gene hits in the pathway and dot color represents the –log_10_ adjusted p-value. **(C)** Average scaled expression of top genes from all cells in Multicellular Program (MP) 1 sorted by expression (columns), across samples plotted with hierarchical clustering (rows) and labeled with clinical variables including body mass index (BMI), age, sex, and measures of glucose intolerance. **(D, E)** Bar plot with relative information flow (left) or overall information flow (right) on the x-axis and predicted ligand-ligand receptor pathways from source cells to target cells. Signaling in HIV+ diabetic is shown in orange while signaling in HIV- diabetic is shown in blue. Pathways with significantly greater interaction in HIV+ diabetics based on Wilcoxon rank sum are labeled in orange while pathways with significantly greater interaction in HIV- diabetics are labeled in blue (p < 0.05). **(D)** Macrophages (source) to all cells (target). **(E)** Stromal (source) to all cells (target). BMI, body mass index; FBG, fasting blood glucose; HbA1c, hemoglobin A1c; IM, intermediate macrophage; LAM, lipid-associated macrophage; NES, normalized enrichment score; PVM, perivascular macrophage.

Transcriptionally, preadipocyte cells were strikingly different with higher expression of genes related to ECM-, genes that impair adipogenesis, and lipid-processing genes in PWH, suggesting impaired adipogenesis and ECM deposition in PWH compared with HIV-negative (**Figures 10A, B**; **Supplementary Table 12**). DIALOGUE uncovered a transcriptionally similar multicellular program and gene expression pattern in diabetic HIV-negative individuals though it included preadipocytes with expression of *BGN, MIF*, and *TIMP1* (**Figure 10C**). The SAT composition and tissue-level transcriptional programs are largely similar between HIV-negative diabetic and diabetic PWH. However, predicted intercellular communication pathways diverged significantly (**Supplementary Figure 11A**). Macrophages from diabetic PWH have increased signaling through APP, resistin, visfatin, and several other pathways that have been associated with macrophage polarization and inflammation (**Figure 10D**). Additionally, analysis of signaling from all cells to macrophages shows increased signaling for several pathways, including IFN, TNF, and IL6 (**Supplementary Figure 11B**). Similarly, the stromal compartment from diabetic PWH had increased signaling to other cells through TGF-β, periostin, chemerin, IL6, and visfatin pathways compared with diabetic HIV-negative persons (**Figure 10E**). Analysis of signaling from all cells to stromal cells showed increased signaling for TNF, IFN, and MIF pathways among diabetic PWH (**Supplementary Figure 11C**). Endothelial cells have an important role in the trafficking of monocytes into tissue (96). Diabetic PWH have greater signaling from endothelial cells to monocytes compared with diabetic HIV-negative persons (**Supplementary Figure 11D**). This includes the fractalkine signaling pathway (CX3C) that has been linked with monocyte infiltration and inflammation (97). Finally, the T cell compartment had increased signaling of TNF, IFN, and several other pathways in diabetic PWH compared with HIV-negative persons (**Supplementary Figure 11E**). Overall, these findings indicate substantial differences in predicted intercellular communication between diabetic PWH and diabetic HIV-negative persons.

## 4 Discussion

In this study, we generated a comprehensive single cell molecular atlas of SAT in PWH to uncover compositional and transcriptional patterns that are associated with glucose intolerance. We showed a shift towards LAM and LAM-like macrophages and T_EM_ cells. Transcriptionally, macrophages shifted from an immunoregulatory M2-like cytokine profile towards a lipid processing phenotype while CD4^+^ and CD8^+^ T cells shifted towards differentiated and effector phenotypes. An expression program in glucose intolerant individuals was defined by upregulation of IFN-γ and TNF-related pathways in CD4^+^ and CD8^+^ T cells, upregulation of lipid-processing genes in macrophages, and increased expression of fibrotic genes in preadipocytes. Intercellular communication analysis predicted increased inflammatory and pro-fibrotic pathways in PWH with glucose intolerance. We further found CD4^+^ memory cells expressing CD69 were most strongly associated with glucose intolerance and alterations in adipocyte gene expression, providing a plausible mechanistic link to altered mature adipocyte function and development of metabolic syndrome (**Supplementary Figure 12**). Finally, as an exploratory analysis, we show that diabetic PWH have a similar compositional profile as HIV-negative individuals, but substantially different predicted intercellular communication pathways skewed towards inflammatory and pro-fibrotic signaling.

Our characterization of SAT resident immune cells and stromal precursor cells expand on previous scRNA-seq descriptions of human adipose tissue in HIV-negative persons (14, 15, 23). Uniquely, we evaluated the role of glucose intolerance in compositional and transcriptional polarization of adipose tissue and leveraged our large cohort size to evaluate the independent contributions of important biological factors including sex, age, and BMI. The macrophage compartment had the greatest compositional differences between non-diabetic and glucose intolerant PWH, which is consistent with prior studies (11, 60, 64, 98–100). LAMs have been previously shown to accumulate with obesity in humans (15), but here we show that they also accumulate with glucose intolerance after adjusting for BMI. LAMs accumulate in crown-like structures surrounding injured adipocytes, and depletion results in greater weight gain, glucose intolerance, and dyslipidemia, suggesting these cells are important for maintaining homeostasis and are likely compensatory in response to adipocyte injury (40). While LAMs identified in this dataset expressed CD11c, we found CD206 surface expression and *MRC1* gene expression was lower relative to PVMs, which was not observed in other studies (15, 40, 60). These differences could be due to differences in cell-sorting strategies and different technologies, and tissue-based analyses should enable better harmonization and clarification of this in the future. Differences in macrophage polarization have been linked to inflammation and obesity in animal studies, although the M1/M2 macrophage phenotypes described in mice are less distinct in human samples (67). Additionally, the transcriptional profile obtained from unstimulated macrophages may not reflect cytokine production under stimulation conditions. Therefore, it is not clear that LAMs are equivalent to M1 macrophages.

There was good agreement among prior datasets for PVMs, which are analogous to M2-like macrophages expressing CD206 (14, 15, 23). We found, similar to other groups, that PVMs express several chemokines and inflammatory genes including *TNF* (15). While these are more abundant in non-diabetic PWH, M2-like macrophages, of which PVM are a subset, have been associated with insulin resistance, mediated in part through TGFβ (101). Of the macrophage subsets, the IM macrophages were most closely linked to myofibroblast proportion and increased with glucose intolerance. These cells had high expression of MHC and CD163 transcripts and shared features with both PVM and LAMs. It is uncertain whether IMs, which have low expression of osteopontin, are a precursor for LAMs or represent a distinct terminal phenotype. Intercellular communication analysis predicts higher signaling through TGFβ and several pro-fibrotic pathways with myofibroblast and preadipocytes in glucose intolerant individuals. Future studies are needed to better define this macrophage cell type.

CD4^+^ tissue-resident cells may also have a key role in the development and maintenance of SAT inflammation. Both multicellular program analysis and intercellular communication show increased IFN and TNF pathways in glucose intolerant PWH, which can promote macrophage polarization and influence adipogenesis (67, 102). Little has been reported on tissue-resident T cells in SAT, though they have a prominent role in regulating responses to infection and contribute to human diseases (103, 104). Our group and others have previously shown CD4^+^ and CD8^+^ tissue-resident T cells accumulate with metabolic disease in PWH (18, 19). This finding is intriguing as SAT CD4^+^ T cells and CD4^+^ tissue-resident T cells in cervical mucosa have been shown to be a reservoir of HIV (17, 105). Future studies will be necessary to determine the mechanism through which tissue-resident T cells affect adipocyte function. Additionally, since CD4 T cells serve as a reservoir for latent HIV infection, these findings may not be true in PWH off ART.

One important question is whether the pathogenesis of metabolic disease is different in PWH compared with HIV-negative persons, which would have implications for preventative and treatment strategies. Our cross-sectional study design and inclusion of only HIV-negative persons with diabetes does not directly address this question. However, we find that although composition and overall global transcriptional pattern is similar regardless of HIV serostatus, diabetic PWH have greater contribution of inflammatory signaling pathways such as IL6, IFN-γ, and TNF compared with HIV-negative persons with diabetes. Signaling to and from endothelial cells was also enhanced in diabetic PWH including CX3CR and CCL pathways. Secreted cytokines from adipose tissue have a profound influence over endothelial inflammation and contribute to endothelial dysfunction (106). Endothelium in turn modulates adipose tissue inflammation, in part through regulation of immune cell migration. These findings suggest targeting adipose tissue infiltration by immune cells may yield beneficial effects in PWH. Dual CCR2/5 antagonists are currently being evaluated in PWH to reduce cardiovascular events, and additional studies to assess the effects on insulin resistance may be warranted (107).

Our study had some limitations. The cross-sectional design precluded an assessment of the temporal course of compositional and transcriptional changes, and future longitudinal scRNA-seq studies are needed. Our separation of participants into three groups by FBG and HbA1c values meant some individuals were on the margin between states, though this was addressed, in part, by our analysis of FBG as a continuous endpoint. Additionally, our cohort lacked a non-diabetic or prediabetic HIV-negative group. Thus, while HIV-negative persons with diabetes appear to have substantially different predicted intercellular communication, further studies incorporating non-diabetic HIV-negative persons will be necessary to understand how baseline differences may impart increased risk of metabolic diseases among PWH. Additionally, despite the size of our unbiased molecular atlas of SAT, we may have missed low-frequency cells that contribute to inflammation. Finally, it is difficult in clinical studies to account for potential confounders. However, we overcame this with reasonably matched groups and a large overall cohort to model the contributions of important biological factors to composition and transcriptional patterns.

In summary, we found unique SAT compositional and transcriptional changes with glucose intolerance and identified a conserved cellular regulatory program that differentiated non-diabetic and glucose intolerant individuals. Our dataset is publicly available to the research community on an interactive platform (http://vimrg.app.vumc.org/). These data provide insight into the complexity and breadth of SAT cells that may contribute to glucose intolerance and accelerate future investigation into the role of stromal and immune cell interactions that may open new avenues of research and lead to the development of therapeutic interventions.

## Data availability statement

The datasets presented in this study can be found in online repositories. The names of the repository/repositories and accession number(s) can be found below: https://www.ncbi.nlm.nih.gov/, GSE198809.

## Ethics statement

The studies involving humans were approved by Vanderbilt University Institutional Review Board. The studies were conducted in accordance with the local legislation and institutional requirements. The participants provided their written informed consent to participate in this study.

## Author contributions

Conceptualization, CeW, JKo, SM. Methodology, CeW, JKo, SM, RG, JS, ChW, JKr, SB. Formal Analysis, SB, CeW, JKo, SK, JKr, FY, RF. Investigation, CeW, ChW, JS, RG, LH. Writing – Original Draft, SB, CeW, JKo, JKr. Writing – Review & Editing, SB, CeW, JKr, JoK, SM, SK, CG, MM, LH, JS, ChW, RF, FY. Funding Acquisition, CeW, JKo, SM. All authors contributed to the article and approved the submitted version.
