# Supplementary material for: Changes in subcutaneous white adipose tissue cellular composition and molecular programs underlie glucose intolerance in persons with HIV
Source: Front Immunol. 2023 Aug 30;14:1152003. doi: 10.3389/fimmu.2023.1152003 (PMC10499182; doi:10.3389/fimmu.2023.1152003)
Supplement: Supplementary file 1 [file DataSheet_1.docx]

Supplementary Material

Changes in Subcutaneous White Adipose Tissue Cellular Composition and Molecular Programs Underlie Glucose Intolerance in Persons with HIV

**Samuel S. Bailin^1^, Jonathan A. Kropski^2,3,4^, Rama D. Gangula^5^, LaToya Hannah^6^, Joshua D. Simmons^5^, Mona Mashayekhi^6^, Fei Ye^7^, Run Fan^7^, Simon Mallal^1,5,8-10^, Christian M. Warren^5^, Spyros A. Kalams^1,5,10^, Curtis L. Gabriel^11^, Celestine N. Wanjalla^1,10,±*^, John R. Koethe^1,3,10,±^**

*** Correspondence:** Celestine N. Wanjalla: celestine.wanjalla@vumc.org

# Supplementary Data

**Key Resources**

| **REAGENT or RESOURCE** | **SOURCE** | **IDENTIFIER** |
| --- | --- | --- |
| **Antibodies** |  |  |
| TotalSeq-C0046 anti-human CD8 Antibody (clone SK1) | BioLegend | Cat#344753 |
| TotalSeq-C0034 anti-human CD3 antibody (clone UCHT1) | BioLegend | Cat#300479 |
| TotalSeq-C0084 anti-human CD56 (NCAM) recombinant antibody (clone QA17A16) | BioLegend | Cat#392425 |
| TotalSeq-C0045 anti-human CD4 antibody (clone SK3) | BioLegend | Cat#344651 |
| TotalSeq-C0050 anti-human CD19 antibody (clone H1B19) | BioLegend | Cat#302265 |
| TotalSeq-C0100 anti-human CD20 antibody (clone 2H7) | BioLegend | Cat#302363 |
| TotalSeq-C0051 anti-human CD14 antibody (clone 63D3) | BioLegend | Cat#367137 |
| TotalSeq-C0083 anti-human CD16 antibody (clone 3G8) | BioLegend | Cat#302065 |
| TotalSeq-C0145 anti-human CD103 antibody (clone BER-ACT8) | BioLegend | Cat#350233 |
| TotalSeq-C0355 anti-human CD137 antibody (clone 4B4-1) | BioLegend | Cat#309839 |
| TotalSeq-C0171 anti-human/mouse/rat CD278 antibody (clone C398.4A) | BioLegend | Cat#313553 |
| TotalSeq-C0146 anti-human CD69 antibody (clone FN50) | BioLegend | Cat#310951 |
| TotalSeq-C0394 anti-human CD71 antibody (clone CY1G4) | BioLegend | Cat#334125 |
| TotalSeq-C0158 anti-human CD134 antibody (Ber-ACT35) | BioLegend | Cat#350035 |
| TotalSeq-C0159 anti-human HLA-DR antibody (clone L243) | BioLegend | Cat#307663 |
| TotalSeq-C0085 anti-human CD25 antibody (clone BC96) | BioLegend | Cat#302649 |
| TotalSeq-C0389 anti-human CD38 antibody (clone HIT2) | BioLegend | Cat#303543 |
| TotalSeq-C0032 anti-human CD154 antibody (clone 24-31) | BioLegend | Cat#310849 |
| TotalSeq-C0006 anti-human CD86 antibody (clone IT2.2) | BioLegend | Cat#305447 |
| TotalSeq-C0170 anti-human CD272 antibody (clone MIH26) | BioLegend | Cat#344527 |
| TotalSeq-C0087 anti-human CD45RO antibody (clone UCHL1) | BioLegend | Cat#304259 |
| TotalSeq-C0148 anti-human CD197 antibody (clone G043H7) | BioLegend | Cat#353251 |
| TotalSeq-C0154 anti-human CD27 antibody (clone O323) | BioLegend | Cat#302853 |
| TotalSeq-C0390 anti-human CD172 antibody (clone A019D5) | BioLegend | Cat#351356 |
| TotalSeq-C0156 anti-human CD95 antibody (clone DX2) | BioLegend | Cat#305651 |
| TotalSeq-C0088 anti-human CD279 antibody (clone EH12.2H7) | BioLegend | Cat#329963 |
| TotalSeq-C0149 anti-human CD161 antibody (clone HP-3G10) | BioLegend | Cat#339947 |
| TotalSeq-C0152 anti-human CD223 antibody (clone 11C3C65) | BioLegend | Cat#369335 |
| TotalSeq-C0151 anti-human CD152 antibody (clone BNI3) | BioLegend | Cat#369621 |
| TotalSeq-C0169 anti-human CD366 antibody (clone F38-2E2) | BioLegend | Cat#345049 |
| TotalSeq-C0160 anti-human CD1c antibody (clone L161) | BioLegend | Cat#331547 |
| TotalSeq-C0250 anti-mouse/human KLRG1 antibody (clone 2F1/KLRG1) | BioLegend | Cat#138433 |
| TotalSeq-C0138 anti-human CD5 antibody (clone UCHT2) | BioLegend | Cat#300637 |
| TotalSeq-C0396 anti-human CD26 antibody (clone BA5b) | BioLegend | Cat#302722 |
| TotalSeq-C0063 anti-human CD45RA antibody (clone HI100) | BioLegend | Cat#304163 |
| TotalSeq-C0139 anti-human TCR gd antibody (clone B1) | BioLegend | Cat#331231 |
| TotalSeq-C0584 anti-human TCR Va24-Ja18 antibody (clone 6B11) | BioLegend | Cat#342925 |
| TotalSeq-C0102 anti-human CD294 antibody (clone BM16) | BioLegend | Cat#350131 |
| TotalSeq-C0581 anti-human TCR Va7.2 antibody (clone 3C10) | BioLegend | Cat#351735 |
| TotalSeq-C0143 anti-human CD196 antibody (clone G034E3 | BioLegend | Cat#353440 |
| TotalSeq-C0179 anti-human CX3CR1 antibody (clone K0124E1) | BioLegend | Cat#355705 |
| TotalSeq-C0071 anti-human CD194 antibody (clone L291H4) | BioLegend | Cat#359425 |
| TotalSeq-C0053 anti-human CD11c antibody (clone S-HCL-3) | BioLegend | Cat#371521 |
| TotalSeq-C0168 anti-human CD57 recombinant antibody (clone QA17A04) | BioLegend | Cat#393321 |
| TotalSeq-C0140 anti-human CD183 antibody (clone G025H7) | BioLegend | Cat#353747 |
| **Software and Algorithms** | | |
| Cell Ranger v6.0.0 | 10X Genomics | N/A |
| Souporcell | (Heaton et al., 2020) | N/A |
| CellChat | (Jin et al., 2021) | N/A |
| SoupX | (Young and Behjati, 2020) | N/A |
| Seurat v4.0.0 | (Stuart et al., 2019) | N/A |
| ClusterProfiler | (Wu et al., 2021) | N/A |
| Slingshot | (Street et al., 2018) | N/A |
| tradeSeq | (Van den Berge et al., 2020) | N/A |
| DoubletFinder | (McGinnis et al., 2019) | N/A |
| PResiduals | (Lui et al., 2020) | N/A |
| Hmisc | (Harrell et al. 2021) | N/A |
| Harmony | (Korsunsky et al., 2019) | N/A |
| MiloR | (Dann et al., 2022) | N/A |
| **Commercial Assays** | | |
| Chromium Single Cell 5’ GEM, Library & Gel Bead Kit, 16 rxns | 10X Genomics | Cat#1000006 |
| Chromium Single Cell 5’ Library Construction Kit, 16 rxns | 10X Genomics | Cat#1000020 |
| Chromium Single Cell 5’ Feature Barcode Library Kit, 16 rxns | 10X Genomics | Cat#1000080 |
| Chromium Chip A Single Cell Kit, 48 rxns | 10X Genomics | Cat#1000152 |
| Chromium i7 Multiplex Kit | 10X Genomics | Cat#120262 |
| Chromium i7 Multiplex Kit N Set A, 96 rxns | 10X Genomics | Cat#1000084 |
| nCounter Inflammation Panel | nanoString | Cat #XT-CSO-HIN2-12 |
| **Chemicals, Peptides, and Recombinant Proteins** | | |
| Collagenase D | Roche | Cat **#**11088866001 |
| Human TruStain FcX | Biolegend | Cat#422302 |

# Supplementary Figures and Tables

## Supplementary Figures

**Supplementary Figure 1. Bioinformatic Pipeline and Quality Metrics. (A)** Flow diagram of bioinformatic pipeline. Cells were called using the Cell Ranger count function with default settings. Soupx was used to remove ambient RNA and the corrected count matrices were used to create a Seurat object. Simultaneously, Souporcell was used to genetically demultiplex the samples. Doublets identified by Souporcell were used as ground truths for DoubletFinder. Next, each Seurat object underwent quality control filtering and downstream dimensional reduction and clustering. The processed objects were then integrated using Harmony correction on the principal components prior to clustering. Cells identified as doublets, clusters with > 60% doublets, or expression of 2 or more major lineages were removed. Cells were subclustered based on major cell lineages and transcriptional doublets were iteratively removed prior to reintegration. **(B)** Bar plot showing each lane (x-axis) and number of cells (y-axis). The graph is split by the total barcodes identified by Cell Ranger (blue) and the cells remaining after doublet and QC removal (green). **(C)** Distribution of read counts per cell (y-axis) by lane (x-axis).  **(D**) Distribution of the number of features (genes) per cell (y-axis) by lane (x-axis). **(E)** Overall integration metric ranking generated by the SCIB pipeline. Unintegrated, harmony integration with feature selection (HVG) and no feature selection, as well as scaled and unscaled were input in the SCIB pipeline. The overall rank was computed by overall batch score (0.4) and bioconservation (0.6) that were scaled for comparison. **(F)** Dot plot with selected genes on the x-axis and cell type on the y-axis. The size of the dot represents the percentage of cells with expression of that gene and the color of the dot represents the average expression level for that gene.

**Supplementary Figure 2. CITE-seq and Pathway Analysis Support Macrophage Annotation. (A)** Antibody derived tags (ADT) for CD11C, CD14, CD1C, and CD16 plotted onto the uniform manifold approximation and projection (UMAP) for myeloid cells. **(B)** UMAP of IM, PVM, Mo-Mac, and LAMs from 59 individuals. **(C)** Macrophage-specific ADT markers for CD9, CD163, CD206, and CD64 plotted onto the UMAP for macrophage cells. **(D)** Gene Ontology (GO) over-representation analysis with macrophage subtype on the x-axis and GO pathway on the y-axis. The size of the circle denotes the number of genes assigned to the pathway while the color denotes the p-value. **(E-H)** Comparison of the current macrophage dataset with annotations from other studies using a gene module score with marker genes that were significantly enriched for each defined cell type for **(E)** Vijay *et. al.,* **(F)** Hildreth *et. al.*, **(G)** Emont *et. al.*, and **(H)** Dick *et. al*. Abbreviations: hMac, human macrophage; IM, intermediate macrophage; IS, immune subset; LAM, lipid-associated macrophage; MHC-II, major histocompatibility complex II; Mo-Mac, monocyte-macrophage; PVM, perivascular macrophage; TLF, *Timd4, Lyve1,* and/or *Folr2*.

**Supplementary Figure 3. Classification of CD4^+^ and CD8^+^ T Cells, and Stromal Cell Subpopulations. (A)** Antibody derived tags (ADT) for CD16, CD27, CD4, CD45RA, CD57, and CD8 plotted onto the uniform manifold approximation and projection (UMAP) for lymphoid cells. **(B**) UMAP of CD4^+^ T cells (n = 8,435 cells) subset from all T cells. **(C)** ADT for CD27, CD45RA, CD57, and CD69, plotted on the CD4^+^ T cell UMAP. **(D**) UMAP of CD8^+^ T cells (n = 11,356 cells) subset from all T cells. **(E)** ADT for CD27, CD45RA, CD57, and CD69, plotted on the CD8^+^ T cell UMAP. **(F)** Scatter plot with proportion of CD4^+^ T_EM_ cells from scRNA-seq on the x-axis and CD4^+^ CD69^+^ cells as a proportion of all CD4^+^ cells from flow cytometry. **(G)** Comparison of the current stromal dataset with annotations from other studies using a gene module score with marker genes that were significantly enriched for each defined cell type. Abbreviations: APC, adipocyte precursor cells; CAP, committed adipose preadipocyte; FIP, fibro-inflammatory progenitor; T_CM_, central memory; T_EM_, effector memory

**Supplementary Figure 4. Cell Type Proportions are Associated with Measures of Glucose Tolerance. (A)** Myeloid differential abundance of each neighborhood group collapsed by cell annotation (y-axis) plotted by log fold change between non-diabetic and glucose intolerant persons with HIV (x-axis). Those with significantly greater abundance or reduced abundance in non-diabetic PWH are plotted in blue and red, respectively. **(B)** Scatter plot with intermediate macrophages as a percent of macrophages on the x-axis and fasting blood glucose (FBG) on the y-axis. The partial Spearman’s ρ is shown. **(C)** Scatter plot with lipid-associated macrophages as a percent of macrophages on the x-axis and hemoglobin A1c (HbA1c) on the y-axis. **(D)** Scatter plot with CD8^+^ T_EM_ cells as a percent of CD8^+^ T cells on the x-axis and FBG on the y-axis. **(E)** Boxplot showing CD4^+^CD69^+^ memory T cells as a percent of total memory CD4^+^ T cells, split by disease state (HIV+ non-diabetic, green; HIV+ prediabetic, blue; HIV+ diabetic, yellow) (n = 21). The horizontal black line represents the median, the box shows the lower and upper quartile limits and the whiskers are 1.5x the interquartile range. Scatter plot with CD4^+^ T_EM_ cells as a percent of CD4^+^ T cells on the x-axis and FBG on the y-axis. * p < 0.05, ** p < 0.01 **(F)** Scatter plot with CD4^+^ T_EM_ cells as a percent of CD4^+^ T cells on the x-axis and FBG on the y-axis. **(G)** Lymphoid differential abundance of each neighborhood group collapsed by cell annotation (y-axis) plotted by log fold change between non-diabetic and glucose intolerant persons with HIV (x-axis). Those with significantly greater abundance or reduced abundance in non-diabetic PWH are plotted in blue and red, respectively. Abbreviations: cDC1, conventional dendritic cell type 1; cDC2B, conventional dendritic cell type 2B, cMo, classical monocyte; ILC, innate lymphoid cell; IM, intermediate macrophage; ISG+ Mo, ISG+ monocyte; LAM, lipid-associated macrophage; mNK, mature natural killer; Migratory DC, migratory dendritic cell; Mo-Mac, monocyte-macrophage; NK, natural killer; nMo, non-classical monocyte; Other Mo, other monocyte; pDC, plasmacytoid dendritic cell; PVM, perivascular macrophage; TCM, T central memory; TEM, T effector memory.

**Supplementary Figure 5. Stromal and Vascular Cell Populations are not Different by Diabetes Status or Measures of Glucose Tolerance. (A)** Boxplot showing stromal fibroblast as a percent of total stromal cells, split by disease state (HIV+ non-diabetic, green; HIV+ prediabetic, blue; HIV+ diabetic, yellow) (n = 59). The horizontal black line represents the median, the box shows the lower and upper quartile limits and the whiskers are 1.5x the interquartile range. * p < 0.05; ns, not significant. **(B)** Partial spearman’s correlations between stromal cell proportions (x-axis) and fasting blood glucose (FBG) or hemoglobin A1c (HbA1c) (y-axis). The area of the circle represents the adjusted p value (larger area = more significant adjusted p-value). Spearman’s ρ is colored red (positive) or blue (negative). **(C)** Boxplot showing vascular cell types as a percent of total vascular cells, split by disease state (HIV+ non-diabetic, green; HIV+ prediabetic, blue; HIV+ diabetic, yellow) (n = 59). **(D**) Partial spearman’s correlations between vascular cell proportions (x-axis) and FBG or HbA1c (y-axis). Abbreviations: EC, endothelial cell; ECM, extracellular matrix; FIB, fibroblast; PreAd, preadipocyte; MyoFib, myofibroblast; ns, not significant; VSMC, vascular smooth muscle cell.

**Supplementary Figure 6. Relationship of Demographic and Select Cell Populations with Composition. (A-C)** Partial spearman’s correlations. Spearman’s ρ for the biological factor (body mass index [BMI] or age) and each cluster proportion was calculated. The area of the circle represents the adjusted p value (larger area = more significant adjusted p-value). Spearman’s ρ is colored red (positive) or blue (negative) for **(A)** myeloid, **(B)** stromal, and **(C)** vascular. **(D)** Partial Spearman’s correlation between T cell proportions and stromal cell proportions. **(E**) Partial Spearman’s correlations between myeloid cell proportions and stromal cell proportions. Abbreviations: BMI, body mass index; cDC1, conventional dendritic cell type 1; cDC2B, conventional dendritic cell type 2B; cMo, classical monocyte; DC, dendritic cell; ECM, extracellular matrix; EC, endothelial cell; Mo, monocyte; nMo, non-classical monocyte; pDC, plasmacytoid dendritic cell; TCM, T central memory; TEM, T effector memory; VSMC, vascular smooth muscle cell.

**Supplementary Figure 7. Transcriptional Profile of Adipose Tissue Macrophage Cells (A)** Slingshot trajectory with monocyte-macrophage 2 as the root and perivascular macrophage as the end cluster embedded on the Uniform Manifold Approximation and Projection (UMAP). **(B)** Ordered and smoothed gene expression (scaled) along the pseudotime trajectory for monocyte-macrophage 2 to perivascular macrophage. Selected genes were significantly differentially expressed along the pseudotime with ≥ log_2_(2) fold change according to TradeSeq. **(C)** Slingshot trajectory with monocyte-macrophage 2 as the root and lipid associated macrophage as the end cluster embedded on the UMAP. **(D)** Ordered and smoothed gene expression (scaled) along the pseudotime trajectory for monocyte-macrophage 2 to lipid associated macrophage. Selected genes were significantly differentially expressed along the pseudotime with ≥ log_2_(2) fold change according to TradeSeq. **(E)** Upset plot for genes with higher expression in prediabetic compared with non-diabetic PWH across cell types. The y-axis shows the set size for each cell type (total number of differentially expressed genes in the cell type) and the x-axis shows the intersection size with other cells (number of shared genes). **(F)** Upset plot for genes with lower expression in prediabetic compared with non-diabetic PWH across cell types. The y-axis shows the set size for each cell type (total number of differentially expressed genes in the cell type) and the x-axis shows the intersection size with other cells (number of shared genes). Abbreviations: EC, endothelial cell; IM, intermediate macrophage; LAM, lipid-associated macrophage; Mo-Mac, monocyte-macrophage; PVM, perivascular macrophage

**Supplementary Figure 8. Multicellular Program Using DIALOGUE Identifies Expression Patterns Associated with Glucose Intolerance.** (**A-E**) Average scaled expression of top genes from multicellular program (MP) 1 sorted by expression (columns), across samples plotted with hierarchical clustering (rows) and labeled with clinical variables including body mass index (BMI), age, sex, and measures of glucose intolerance. (**A**) Perivascular macrophages. (**B**) Monocyte-macrophage 1. (**C**) Intermediate macrophages. (**D**) Myofibroblast. (**E**) All cells in MP1. Abbreviations: BMI, body mass index; FBG, fasting blood glucose; HbA1c, hemoglobin A1c.

**Supplementary Figure 9. Intercellular Communication is Different Between Glucose Intolerant and Non-diabetic PWH.** (**A-F**) Bar plot with relative information flow (left) or overall information flow (right) on the x-axis and predicted ligand-ligand receptor pathways from source cells to target cells. Signaling with glucose intolerance is shown in orange while signaling in non-diabetics is shown in blue. Pathways with significantly greater interaction with glucose intolerance based on Wilcoxon rank sum are labeled in orange while pathways with significantly greater interaction in non-diabetic are labeled in blue (p < 0.05). (**A**) Lipid-associated macrophages (LAMs) (source) to myofibroblast and cycling myofibroblast (target). (**B**) LAMs (source) to preadipocytes and progenitor cells (target). (**C**) Myofibroblast and cycling myofibroblast (source) to LAMs. (**D**) Preadipocytes and progenitor cells (source) to LAMs (target). (**E**) CD4^+^ and CD8 T effector memory cells (source) to IM and LAMs (target). (**F**) IM and LAMs (source) to CD4^+^ and CD8 T effector memory cells.

**Supplementary Figure 10. The Relationship of Cell Proportions in HIV-negative Diabetic Individuals and Body Mass Index or Age.** (**A**) Overall uniform manifold approximation and projection of all cells, split by disease status (HIV+ non-diabetic, green; HIV+ prediabetic, blue; HIV+ diabetic, yellow; HIV- diabetic, orange). (**B-E**) Partial spearman’s correlations. Spearman’s ρ for the biological factor (body mass index [BMI] or age) and each cluster proportion was calculated. The area of the circle represents the adjusted p value (larger area = more significant adjusted p-value). Spearman’s ρ is colored red (positive) or blue (negative) for (**B**) CD4^+^ T cells, (**C**) CD8^+^ T cells (**D**) Myeloid, and (**E**) Lymphoid. Abbreviations: BMI, body mass index; cMo, classical monocyte; cDC1, conventional dendritic cell type 1; cDC2B, conventional dendritic cell 2B; DC, dendritic cell; ILC, innate lymphoid cell; mNK, mature natural killer; Mo-Mac, monocyte-macrophage; MT, metallothionein; nMo, non-classical monocyte; PVM, perivascular macrophage; pDC, plasmacytoid dendritic cell; TCM, central memory; TEM, effector memory.

**Supplementary Figure 11. HIV-negative Diabetes Have Divergent Cell-Cell Communication Signaling Compared with HIV+ Diabetics.** (**A**) Relative number of interactions (left) and strength of interactions (right) comparing PWH with glucose intolerance and HIV-negative persons with diabetes. Increased and decreased relative number of strength of interactions are shown in red and blue, respectively. The target cells are shown on the x-axis and the source cells are shown on the y-axis. Rows and columns were plotted with hierarchical clustering. (**B-E**) Bar plot with relative information flow (left) or overall information flow (right) on the x-axis and predicted ligand-ligand receptor pathways from source cells to target cells. Signaling in HIV+ diabetic is shown in orange while signaling in HIV- diabetic is shown in blue. Pathways with significantly greater interaction in HIV+ diabetics based on Wilcoxon rank sum are labeled in orange while pathways with significantly greater interaction in HIV- diabetics are labeled in blue (p < 0.05). (**B**) All cells (source) to macrophages (target). (**C**) All cells (source) to stromal cells (target). (**D**) Endothelial cells (source) to monocytes (target) (**E**) T cells (source) to all cells (target). Abbreviations: Adipose Prog, adipose progenitors; cMo, classical monocyte; cDC1, conventional dendritic cell type 1; cDC2B, conventional dendritic cell type 2B; DC, dendritic cell; EC, endothelial cell; FIB, fibroblast; ILC, innate lymphoid cell; IM, intermediate macrophage; ISG+, interferon-stimulated gene +; LAM, lipid-associated macrophage; Mac, macrophage; mNK, mature natural killer; Mo, monocyte; MT, metallothionein+; myoFIB, myofibroblast; NK, natural killer; nMo, non-classical monocyte; pDC, plasmacytoid dendritic cell; PreAd, preadipocyte; PVM, perivascular macrophage; T_CM_, T central memory; T_EM_, T effector memory.

**Supplementary Figure 12. Theoretical Model for the Compositional and Transcriptional Shift in Persons with HIV with Glucose Intolerance.** In persons with HIV (PWH) who are metabolically healthy, subcutaneous adipose tissue (SAT) preadipocyte populations expressing markers of adipogenesis accompanied by immunoregulatory perivascular macrophages (PVMs) and CD4^+^ naïve T cells may contribute to a pattern of immunoregulation and reduced inflammation. With the development of glucose intolerance, injury to adipocytes due to obesity, HIV, and anti-retroviral therapy result in SAT adipocyte cell necrosis, polarization of macrophages towards lipid-metabolism, and T cells towards an inflammatory CD4^+^ T_EM_ CD69^+^ tissue memory resident that promote a coordinated inflammatory response in SAT that contributes to reduced adipogenesis and markers of fibrosis.

## Supplementary Tables

**Supplementary Table 1.** **RNA and Antibody-Derived Tag (ADT) Quality Metrics.** Descriptive summary derived from Cell Ranger for RNA and ADT by lane.

**Supplementary Table 2.** **Souporcell Summary Metrics.** Summary of ambient RNA (%) estimate, singlet and doublet assignment, and genotypic cluster assignments per lane.

**Supplementary Table 3.** **Individual Cell Contributions.** Summary of the number of cells per cell type by individual.

**Supplementary Table 4.** **Canonical Gene Markers.** Table of genes used to manually validate cell identities.

**Supplementary Table 5. Differential Gene Expression for Each Cell Type.** Differentially expressed genes by each cell type using the entire dataset.

**Supplementary Table 6. Differential Gene Expression for Each Major Cell Type.** Differentially expressed genes for each major cell type (vascular, stromal, lymphoid, and myeloid).

**Supplementary Table 7.** **Differential Gene Expression for CD4 and CD8 T Cells, and Macrophages.** Differentially expressed genes by CD4 type, C8 type, and macrophage subtype.

**Supplementary Table 8.** **Pseudobulk Differential Gene Expression Between Prediabetic and Non-diabetic PWH.** Differentially expressed genes by pseudobulk method (DESeq2) between prediabetic and non-diabetic PWH between select cell types of interest, adjusted for age, sex, and body mass index.

**Supplementary Table 9.** **Pseudobulk Differential Gene Expression Between Diabetic and Non-diabetic PWH.** Differentially expressed genes by pseudobulk method (DESeq2) between diabetic and non-diabetic PWH between select cell types of interest, adjusted for age, sex, and body mass index.

**Supplementary Table 10. Relationship of CD4^+^ CD69^+^ T Cells by flow (sheet 1) and CD4^+^ T_EM_ Cells by single-cell RNA sequencing and 77 adipocyte-related genes.** A linear model was fit with the log_2_-transformed gene count as the dependent variable and the CD4 cell proportion as the independent variable adjusted for age, sex, body mass index, diabetes status, and batch.

**Supplementary Table 11.** **Characteristics for HIV- persons with diabetes.**

**Supplementary Table 12.** **Pseudobulk Differential Gene Expression Between diabetic PWH and diabetic HIV-negative persons.** Differentially expressed genes by pseudobulk method (DESeq2) between diabetic PWH and diabetic HIV-negatie persons between select cell types of interest, adjusted for age, sex, and body mass index.
